# Rosaceae, Brassicaceae and pollen beetles: exploring relationships and evolution in an anthophilous beetle lineage (Nitidulidae, *Meligethes-*complex of genera) using an integrative approach

**DOI:** 10.1186/s12983-021-00390-4

**Published:** 2021-03-06

**Authors:** Meike Liu, Min Huang, Andrew Richard Cline, Emiliano Mancini, Andrea Scaramuzzi, Simone Paradisi, Paolo Audisio, Davide Badano, Simone Sabatelli

**Affiliations:** 1 Key Laboratory of Plant Protection Resources and Pest Management of Ministry of Education, Entomological Museum, Northwest A&F University, Yangling, Xianyang, Shaanxi China; 2 College of Agriculture, Yangtze University, Jingzhou, 434025 Hubei China; 3 California Department of Food & Agriculture, Plant Pest Diagnostics Center, Sacramento, CA USA; 4 Dipartimento di Biologia e Biotecnologie “Charles Darwin”, Sapienza Università di Roma, Rome, Italy

**Keywords:** Pollen beetles, Rosaceae, Brassicaceae, Evolution, Host-shift, Palaearctic region

## Abstract

**Background:**

*Meligethes* are pollen-beetles associated with flowers of Rosaceae as larvae. This genus currently consists of 63 known species in two subgenera, *Meligethes* and *Odonthogethes,* predominantly occurring in the eastern Palaearctic. We analyzed 74 morphological and ecological characters (169 states) of all species, as well as of 11 outgroup species from 7 Meligethinae genera (including *Brassicogethes*), to investigate their phylogeny. We also conducted a parallel molecular analysis on 9 *Meligethes*, 9 *Odonthogethes*, 3 *Brassicogethes* and 2 *Meligethinus* species based on DNA sequence data from mitochondrial (COI, 16S) and nuclear (CAD) genes.

**Results:**

Morphological phylogenetic reconstructions supported the monophyly of the whole genus and clades corresponding to purported subgenera *Meligethes* s.str. and *Odonthogethes.* Main species-groups were mostly confirmed, however some unresolved polytomies remained. Molecular data placed members of *Brassicogethes* (including 42 mostly W Palearctic species associated with Brassicaceae) as sister to *Odonthogethes,* with this clade being sister to *Meligethes* s.str. This phylogenetic scenario suggests that monophyletic *Meligethes* s.str., *Odonthogethes* and *Brassicogethes* should be regarded alternatively as three subgenera of a monophyletic *Meligethes*, or three genera in a monophyletic genus-complex, with mutually monophyletic *Brassicogethes* and *Odonthogethes*. Molecular analyses estimated the origin of this lineage at ca. 14–15 Mya from a common stem including *Meligethinus*.

**Conclusions:**

We hypothesize that the ancestor of *Meligethes* specialized on Rosaceae in the Middle Miocene (likely in Langhian Age) and subsequently radiated during Late Miocene and Plio-Pleistocene maintaining a trophic niche on this plant family. This radiation was primarily due to geographic isolation in E Asiatic mountain systems. Combined evidence from morphology, ancestral state parsimony reconstruction of host-plant associations and molecular evidence suggested that Rosoideae (*Rosa* spp.) represented the ancestral hosts of *Meligethes* s.str., followed by an independent shift of ancestral *Odonthogethes* (ca. 9–15 Mya) on *Rubus* (Rosoideae) and members of Rosaceae Spiraeoideae. Other ancestral *Odonthogethes* probably shifted again on the unrelated plant family Brassicaceae (maybe 8–14 Mya in S China), allowing a rapid westward radiation of the *Brassicogethes* clade.

**Supplementary Information:**

The online version contains supplementary material available at 10.1186/s12983-021-00390-4.

## Background

**Fig. 1:**
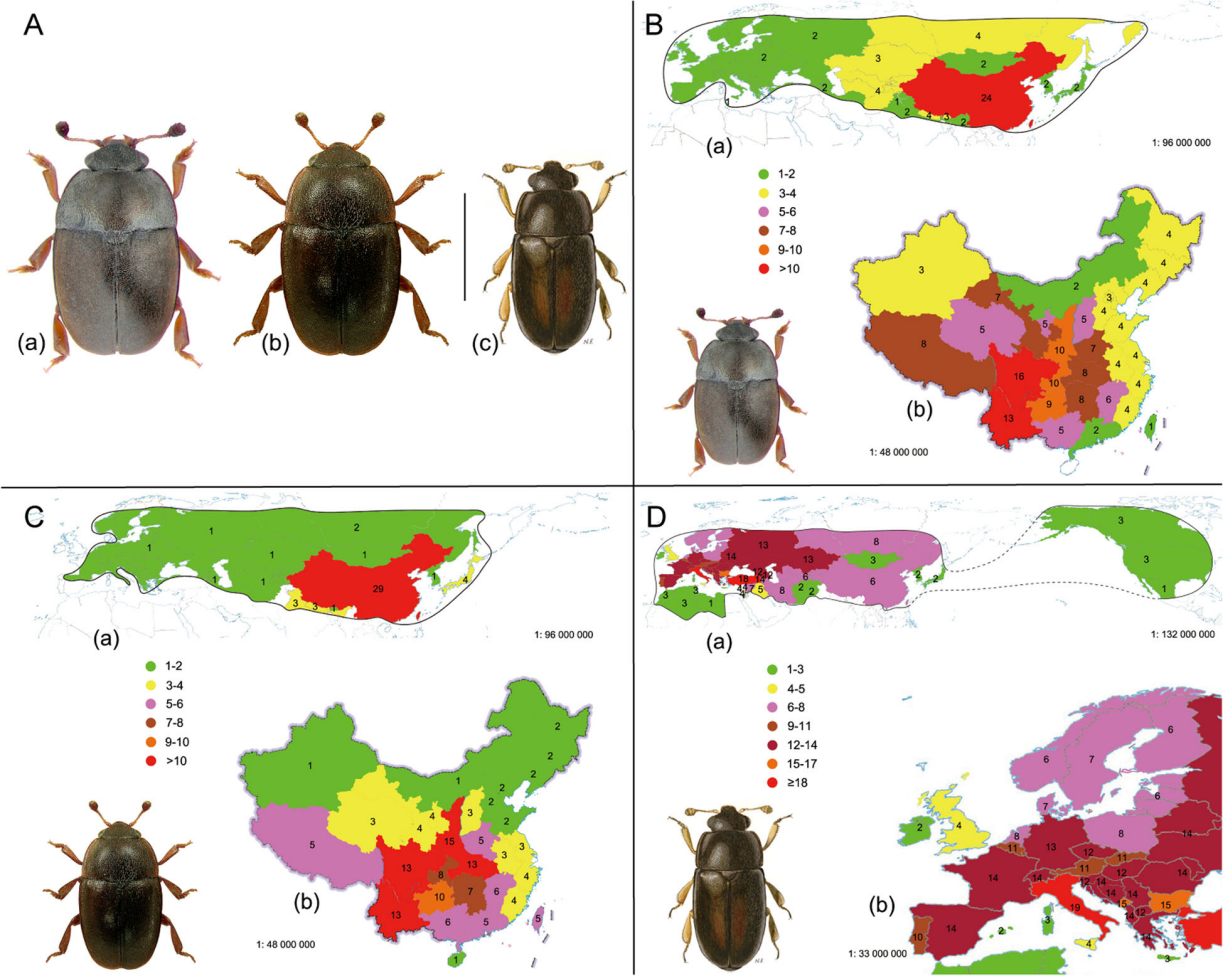
**a** *Meligethes* complex of genera; (a) *Meligethes flavimanus* Sturm, 1845 from Russia, E Siberia (photo by Kirill Makarov); (b) *Odonthogethes denticulatus* (Heer, 1841) from Poland (photo by Lech Borowiec); (c) *Brassicogethes longulus* (Schilsky, 1894) from E Turkey (color drawing by Niccolò Falchi)*.* Scale bar = 1.5 mm. **b** (a) Map of *Meligethes* s.str. Species richness throughout their overall geographic range. Geographic units are all defined political countries, except for the Asiatic portion of the Russian Federation (the main geographic subdivisions are considered separately), and China (all China is represented). European and Caucasian countries, where two widespread species are present, are considered together. (b) Map of *Meligethes* s.str. Species richness in China (each administrative province and Taiwan are considered separately). **c** (a) Map of *Odonthogethes* species richness throughout their overall geographic range [same criteria as in (**b**)(a)]. European and Caucasian countries, where only one widespread species is present, are considered together. (b) Map of *Odonthogethes* species richness in China. **d** (a) Map of *Brassicogethes* species richness throughout their overall geographic range [same criteria as in (**b**)(a)]. Nearctic countries are considered together. (b) Map of *Brassicogethes* species richness in Europe and in Mediterranean areas (each country and the main, distinct geographical units are considered separately). Numbers of each *Meligethes*, *Odonthogetes* and *Brassicogethes* species are reported for each country, main geographic subdivision or administrative unit. Countries with absence of the studied species are represented by no color

Nitidulidae, with almost 4500 known species, is a mid-sized family of the order Coleoptera. Within this taxon, the diverse subfamily Meligethinae comprises about 700 pollen-eating species described worldwide [1–4]. Recently, the classification of Meligethinae underwent numerous changes in light of both molecular and morphological evidence, restricting the concept to several genera, particularly in the case of the previously polyphyletic genus *Meligethes* Stephens, 1830 [2, 5, 6]. *Meligethes*, even as presently bounded [2] [Fig. 1a (a), (b)], is still a rather species-rich genus, consisting of more than 60 species predominantly occurring in the Eastern Palaearctic [7–10] (Fig. 1 b-c; Table 1). This group includes species that are all associated with flowers of Rosaceae as larvae [2]. Most of *Meligethes* are oligophagous, although some members appear strictly monophagous [1, 9, 10]. Some locally common *Meligethes* [e.g. *M. atratus* (Oliver, 1790), *M. flavimanus* Stephens, 1830, *M. violaceus* Reitter, 1873)] also represent economically significant potential pests, attacking blossoms of ornamental roses (*Rosa* spp.) and plum trees (*Prunus* spp.) in Europe, Japan, and elsewhere [1, 11–13].

**Table 1:** Summary of information on all 62 described species of *Meligethes* s.str. and *Odonthogethes*

specific epithet	author(s) and year of description	subgenus	species group	host- plant	distribution	type(s)	notes
*argentithorax*	Audisio, Sabatelli & Jelínek, 2015	*Meligethes*	*auripilis/* *binotatus*	*Rosa* spp.	W and SW China “http://encyclopedia2.thefreedictionary.com/Qinghai“(Sichuan)	CAR-MZUR	
*atratus*	(Olivier, 1790)	*Meligethes*	*atratus*	*Rosa* spp.	W Palaearctic Region, N China, Russia	MHNP	
*aurantirugosus*	Audisio, Sabatelli & Jelínek, 2015	*Odonthogethes*	*aurantirugosus*	*Rubus* sp.?	W Nepal	IZAS	female unknown
*aureolineatus*	Audisio, Sabatelli & Jelínek, 2015	*Meligethes*	*auripilis/ binotatus*	*Rosa* spp.	SW China (Sichuan)	NMPC	female genitalia unknown
*auricomus*	Rebmann, 1956	*Meligethes*	*auripilis/ binotatus*	*Rosa* sp.?	SE China (Fujian, Jiangxi)	SMF	
*aurifer*	Audisio, Sabatelli & Jelínek, 2015	*Meligethes*	*auripilis/ binotatus*	*Rosa* spp.	Central China (Shaanxi, Shanxi)	NMPC	
*auripilis*	Reitter, 1889	*Meligethes*	*auripilis/ binotatus*	*Rosa* spp.	SW and NW China (Sichuan, Yunnan, Gansu, Shanxi, Shaanxi)	unknown	
*auropilosus*	Liu, Yang, Huang, Jelínek & Audisio, 2016	*Meligethes*	*nepalensis*	*Rosa* spp.?	SW and Central China (Xizang, Sichuan, Hubei, Shaanxi)	IZAS	
*aurorugosus*	Liu, Yang, Huang, Jelínek & Audisio, 2016	*Odonthogethes*	*aurantirugosus*	*Rubus* sp.?	W China (Xizang)	IZAS	
*binotatus*	Grouvelle, 1894	*Meligethes*	*auripilis/ binotatus*	*Rosa* spp.	NE India, Nepal, SW China (Yunnan, Sichuan), N Myanmar, Bhutan	MHNP	
*bourdilloni*	Easton, 1968	*Odonthogethes*	*chinensis*	*Rubus* sp.?	E Nepal	BMNH	
*brassicogethoides*	Audisio, Sabatelli & Jelínek, 2015	*Odonthogethes*	*chinensis*	*Rubus* sp.?	SW China (Yunnan)	NMPC	
*castanescens*	Grouvelle, 1903	*Odonthogethes*	*ferrugineus*	unknown	N India (Darjeeling), SW China (Yunnan)	MHNP	
*chinensis*	Kirejtshuk, 1979	*Odonthogethes*	*chinensis*	*Rubus* spp.	W and Central China (Xizang, Yunnan, Sichuan, Chongqing, Gansu, Shaanxi, Henan, Hubei)	ZIN	
*cinereoargenteus*	Audisio, Sabatelli & Jelínek, 2015	*Meligethes*	*auripilis/ binotatus*	*Rosa* spp.	SW China (Sichuan)	NMPC	
*cinereus*	Jelínek, 1978	*Meligethes*	*nepalensis*	*Rosa* sp.?	Bhutan	NHMB	
*clinei*	Audisio, Sabatelli & Jelínek, 2015	*Meligethes*	*auripilis/ binotatus*	*Rosa* sp.?	SW China (Yunnan)	CAS	female unknown
*cyaneus*	Easton, 1957	*Meligethes*	*atratus*	*Rosa* sp.?	Japan	BMNH	
*denticulatus*	(Heer, 1841)	*Odonthogethes*	*denticulatus*	*Rubus* spp.	W Palaearctic Region, N China, Russia	ETHZ	
*elytralis*	Audisio, Sabatelli & Jelínek, 2015	*Meligethes*	*auripilis/ binotatus*	*Rosa* spp.	SW China (Sichuan)	NMPC	
*ferrugineus*	Reitter, 1873	*Odonthogethes*	*ferrugineus*	unknown	N India (Sikkim)	MHNP	
*ferruginoides*	Audisio, Sabatelli & Jelínek, 2015	*Odonthogethes*	*ferrugineus*	*Pyracantha* sp.	Central and SW China (Hubei, Sichuan)	NMPC	
*flavicollis*	Reitter, 1873	*Odonthogethes*	*flavicollis*	*Photinia* sp.?	E Russia, Japan, North Korea, SW, SE and Central China (Henan, Zhejiang, Chongqing, Jiangxi, Taiwan)	BMNH	
*flavimanus*	Stephens, 1830	*Meligethes*	*atratus*	*Rosa* spp.	W Palaearctic Region, N China, Russia	BMNH	
*griseus*	Jelínek, 1978	*Meligethes*	*nepalensis*	*Rosa* sp.?	Bhutan	NHMB	
*hammondi*	Kirejtshuk, 1980	*Meligethes*	*atratus*	*Rosa* spp.	W and central China (Shaanxi, Sichuan, Shanxi, Henan, Hubei)	BMNH	
*henan*	Audisio, Sabatelli & Jelínek, 2015	*Odonthogethes*	*chinensis*	*Rubus* sp.?	Central China (Henan)	NMPC	
*inexpectatus*	Liu, Huang, Cline, Sabatelli & Audisio, 2017	*Odonthogethes*	*chinensis*	*Rubus* sp.?	SW China (Sichuan)	NWAU	
*lloydi*	Easton, 1968	*Odonthogethes*	*pectoralis*	*Malus* sp.?	Nepal, SW China (Yunnan)	BMNH	
*luteomaculatus*	Liu, Huang, Cline & Audisio, 2018	*Odonthogethes*	*chinensis*	*Rubus* sp.?	Central China (Hubei)	NWAU	
*luteoornatus*	Audisio, Sabatelli & Jelínek, 2015	*Odonthogethes*	*chinensis*	*Rubus* sp.?	SW China (Yunnan)	CAS	
*lutra*	Solsky, 1876	*Meligethes*	*vulpes*	*Rosa* spp.	Uzbekistan	ZMUM	
*macrofemoratus*	Liu, Yang, Huang, Jelínek & Audisio, 2016	*Meligethes*	*auripilis/ binotatus*	*Rosa* spp.	Central China (Ningxia, Shaanxi, Hubei)	MHBU	
*marmota*	Audisio, Sabatelli & Jelínek, 2015	*Meligethes*	*auripilis/ binotatus*	*Rosa* sp.?	Nepal	MHNG	
*martes*	Audisio, Sabatelli & Jelínek, 2015	*Meligethes*	*vulpes*	*Rosa* sp.?	SW and N China (Shaanxi, Shanxi, Sichuan)	NMPC	
*melleus*	Grouvelle, 1908	*Meligethes*	*vulpes*	*Rosa* spp.	Myanmar, N India, N Pakistan, Afghanistan, S Tajikistan, Nepal	MNHN	
*nepalensis*	Easton, 1968	*Meligethes*	*nepalensis*	*Rosa* spp.	Nepal, N India	BMNH	
*nigroaeneus*	Audisio, Sabatelli & Jelínek, 2015	*Odonthogethes*	*chinensis*	*Rubus* sp.?	SW China (Yunnan)	CAS	
*nivalis*	Audisio, Sabatelli & Jelínek, 2015	*Meligethes*	*auripilis/ binotatus*	*Rosa* sp.?	SW China (Xizang, Yunnan, Chongqing)	NMPC	
*occultus*	Audisio, Sabatelli & Jelínek, 2015	*Odonthogethes*	*chinensis*	*Rubus* sp.?	SW China (Yunnan)	NMPC	male unknown
*pallidoelytrorum*	Chen & Kirejtshuk, 2013	*Odonthogethes*	*chinensis*	*Rubus* sp.	SW China (Sichuan)	IZAS	
*pectoralis*	Rebmann, 1956	*Odonthogethes*	*pectoralis*	*Malus* sp.	S Japan, SW, SE and Central China (Guizhou, Hubei, Fujian, Zhejiang, Taiwan)	SMF	
*pseudochinensis*	Audisio, Sabatelli & Jelínek, 2015	*Odonthogethes*	*chinensis*	*Pyracantha* sp.	Central China (Chongqing, Shaanxi, Hubei)	NMPC	
*pseudopectoralis*	Audisio, Sabatelli & Jelínek, 2015	*Odonthogethes*	*pectoralis*	*Malus* sp.?	SW China (Yunnan, Sichuan)	NMPC	
*sadanarii*	S.-T. Hisamatsu, 2009	*Odonthogethes*	*pectoralis*	*Malus* sp.?	SE China (Taiwan)	MNST	
*schuelkei*	Audisio, Sabatelli & Jelínek, 2015	*Odonthogethes*	*chinensis*	*Rubus* sp.?	W China (Sichuan, Shaanxi?)	NMPC	
*scrobescens*	Chen, Lin, Huang & Yang, 2015	*Odonthogethes*	*chinensis*	*Rubus* sp.?	SW China (Sichuan, Hubei, Chongqing)	IZAS	
*semenovi*	Kirejtshuk, 1979	*Meligethes*	*auripilis/ binotatus*	*Rosa* spp.	SE Russia (Ussuri), Central, SW and NW China (Sichuan, Hubei, Shaanxi)	ZIN	
*shirakii*	S. Hisamatsu, 1956	*Odonthogethes*	*ferrugineus*	*Prunus* sp.?	S Japan (Kyū-Shū), SE China (Guizhou, Zhejiang, Taiwan)	EUMJ	
*simulator*	Audisio, Sabatelli & Jelínek, 2015	*Odonthogethes*	*chinensis*	*Rubus* sp.?	Central China (Gansu, Shaanxi)	NMPC	
*stenotarsus*	Audisio, Sabatelli & Jelínek, 2015	*Meligethes*	*auripilis/ binotatus*	*Rosa* sp.?	SW China (N Yunnan, Xizang)	NKMS	
*torquatus*	Jelínek, 1997	*Meligethes*	*atratus*	*Rosa* spp.	SE China (Taiwan)	NMPC	
*transmissus*	Kirejtshuk, 1988	*Meligethes*	*auripilis/ binotatus*	*Rosa* spp.	SW China (Sichuan, Yunnan)	ZIN	
*trapezithorax*	Liu, Huang, Cline & Audisio, 2018	*Odonthogethes*	*chinensis*	*Rubus* sp.?	Central China (Hubei)	NWAU	
*tricuspidatus*	Liu, Huang, Cline & Audisio, 2018	*Odonthogethes*	*chinensis*	*Rubus* sp.?	Central China (Hubei)	NWAU	
*tryznai*	Audisio, Sabatelli & Jelínek, 2015	*Meligethes*	*auripilis/ binotatus*	*Rosa* spp.	SW China (Yunnan/ Xizang border)	NMPC	
*violaceus*	Reitter, 1873	*Meligethes*	*atratus*	*Rosa* spp.	China (Anhui, Shaanxi, Hubei, Zhejiang, Fujian, Guizhou, Jiangxi, Yunnan, Sichuan), SE Russia (Ussuri), Japan	BMNH	
*volkovichi*	Audisio, Sabatelli & Jelínek, 2015	*Meligethes*	*nepalensis*	*Rosa* sp.?	SW China (Yunnan)	CAS	
*vulpes*	Solsky, 1876	*Meligethes*	*vulpes*	*Rosa* spp.	Uzbekistan, Kyrgyzstan, Tajikistan, Turkmenistan, NW China (Xinjiang)	ZMUM	
*wagneri*	Rebmann, 1956	*Odonthogethes*	*denticulatus*	*Sorbaria* sp.?	SE and Central China (Fujian, Zhejiang, Taiwan, E Shaanxi)	SMF	
*xenogynus*	Audisio, Sabatelli & Jelínek, 2015	*Odonthogethes*	*ferrugineus*	*Rubus* sp.?	SW and central China (Sichuan, Shaanxi)	NMPC	
*yak*	Liu, Yang, Huang, Jelínek & Audisio, 2016	*Meligethes*	*auripilis/ binotatus*	*Rosa* sp.?	SW China (Sichuan)	IZAS	female unknown

The closely related and purported sister genus *Brassicogethes* Audisio & Cline, 2009 [Fig. 1 a (c), d], comprises instead some forty, mostly Western Palaearctic species, all associated with Brassicaceae [2, 5, 14–23], and a few species [e.g. *B. aeneus* (F.) and *B. viridescens* (F.)] represent economically important pests, massively attacking blossoms of oilseed rapes, broccoli, cauliflowers, and others.

Most species of *Meligethes* (and several within the related genus *Brassicogethes*) have been recently analyzed through an integrated approach combining morphological, molecular, and bionomical data on larval ecology [2, 5, 14–25]. These contributions were based on morphological and molecular data from adults and on larval bionomical data. The thus far available molecular data set for *Meligethes* and *Brassicogethes,* including sequences of three mitochondrial and nuclear genes for 23 species (Table 2), allowed depicting a first phylogenetic scenario of *Meligethes* and some other related genera, and also provided a framework for understanding the origin of this group of Meligethinae, its evolution on different subfamilies of Rosaceae, and, possibly, the shift of ancestral *Brassicogethes* to Brassicaceae.

**Table 2:** Species and specimens of [*Meligethes* s.str. and *Odonthogethes*], *Brassicogethes* and *Meligethinus* used for molecular analysis. GenBank accession numbers for each sample and location are also reported

***Species***	***Sample ID***	***Localities***	***COI***	***16S***	***CAD***
*Meligethes auripilis*	MAU1_1	China- Sichuan, Kangding co	MT949505	MT957159	MT966856
*Meligethes auripilis*	MAU1_2	China- Sichuan, Kangding co	MT949506	MT957160	MT966857
*Meligethes auropilosus*	MAURO1_1	China- Hubei, Shennongjia Forest, Shennong Peak area	MT949504	MT957158	MT966855
*Meligethes binotatus*	MBI1_1	China- Sichuan-Xiangcheng co.	MT949503	MT957157	MT966854
*Meligethes chinensis*	4C2	China- Chongqing, Shizhu, Huangshui	MT949517	MT957171	MT966865
*Meligethes elytralis*	MEL1_1	China- Sichuan, Xiangcheng co	MT949509	MT957163	MT966860
*Meligethes ferruginoides*	MFE1_1	China- Sichuan-Moxi-Yanzigou	MT949511	MT957165	/
*Meligethes hammondi*	MHA 1_1	China- Hubei, Shennongjia forest, Muyu	MT949502	MT957156	MT966853
*Meligethes luteomaculatus*	MLU1_1	China- Hubei, Shennongjia Forest, Shennong Peak area	MT949518	MT957172	MT966866
*Meligethes macrofemoratus*	MMA1_1	China- Hubei, Shennongjia forest	MT949510	MT957164	MT966861
*Meligethes pallidoelytrorum*	MPA2_1	China-Sichuan-Ganzi-Moxi Town	MT949521	MT957175	MT966869
*Meligethes pectoralis*	MPEC1_1	China- Guizhou-Tongzi-Louguan-Mts-Wanmuhuahai	MT949515	MT957169	/
*Meligethes pectoralis*	MPEC1_2	China- Guizhou-Tongzi-Louguan-Mts-Wanmuhuahai	MT949514	MT957168	/
*Meligethes pseudochinensis*	4A10	China- Hubei, Shennongjia forest, Muyu	MT949523	MT957177	MT966871
*Meligethes pseudochinensis*	4C5	China- Chongqing, Shizhu, Huangshui	MT949522	MT957176	MT966870
*Meligethes semenovi*	2C41	China- Tibet, Shannan, Cuona, Gongri	MT949508	MT957162	MT966859
*Meligethes semenovi*	2C42	China- Tibet, Shannan, Cuona, Gongri	MT949507	MT957161	MT966858
*Meligethes scrobescens*	4B7	China- Chongqing, Shizhu, Huangshui	MT949516	MT957170	MT966864
*Meligethes transmissus*	MTR1_1	China- Sichuan, Kangding co	MT949501	MT957155	MT966852
*Meligethes violaceus*	MVI1_1	China- Zhejiang-Quzhou City-Jiangshan City	MT949519	MT957173	MT966867
*Meligethes wagneri*	4C4	China- Chongqing, Shizhu, Huangshui	MT949520	MT957174	MT966868
*Meligethes xenogynus*	3A31	China- Shaanxi, Meixian, Haoping temple	MT949512	MT957166	MT966862
*Meligethes xenogynus*	3A32	China- Shaanxi, Meixian, Haoping temple	MT949513	MT957167	MT966863
*Brassicogethes coracinus*	CR8_1	Turkey-Ardahan-road between Göle and Susuz	MT949498	MT957152	MT966849
*Brassicogethes aeneus*	BAE13_3	Italy- Lazio- Pomezia - Borgo di pratica di mare	MT949496	MT957150	MT966847
*Brassicogethes aeneus*	BAE13_4	Italy- Lazio- Pomezia - Borgo di pratica di mare	MT949497	MT957151	MT966848
*Brassicogethes salvan*	BSA1.1	Italy- Piemonte- Mt.Palafrè- Lago inf. Del Frisson	MT949495	MT957149	MT966846
*Meligethinus peringueyi*	MEP1_1	Mozambique- Maputo,Reserva Especial	MT949500	MT957154	MT966851
*Meligethinus dolosus*	MED1_2	Mozambique- Maputo,Reserva Especial	MT949499	MT957153	MT966850

Although the genus *Meligethes* was included in previous phylogenetic studies, those were aimed to resolve suprageneric classification using molecular approaches [24–26] and only a few widespread W-Palaearctic species were represented. Herein, we analyzed 69 morphological characters (with 157 character states) of adults (Fig. 5) for all 63 known *Meligethes* (s.l.) species (including a thus far undescribed species from S China), as well as 11 outgroup species belonging to 7 different related Meligethinae genera, including 3 *Brassicogethes* species. Five additional bionomical characters (with 12 character states) were also analyzed combining available (published and unpublished) data on *Meligethes* larval ecology. The present work, then, constitutes the first comprehensive phylogenetic analysis at the species level of this large group, with a focus on E Palaearctic lineages.

### The genus *Meligethes* Stephens, 1830

As recently summarized [7], the genus *Meligethes* [Fig. 1 a (a), (b)] was formally established by Stephens in 1830 [27], based on the type species *Nitidula rufipes* Marsham, 1802 [present day valid name: *Meligethes atratus* (Olivier, 1790)]. A new preliminary phylogenetic scenario for *Meligethes* s.l., previously including a heterogeneous and polyphyletic mixture of taxa, formally comprising more than 500 species worldwide, was recently presented [2, 24–26]. In the former paper [2], which was based on morphology of adults and preliminary molecular data, 22 genera were described as new, and 6 previously recognized subgenera of *Meligethes* were elevated to generic rank, delimiting *Meligethes* to some thirty Palaearctic species that utilized Rosaceae as larval host-plants. Following the original descriptions of the few European species [27–30], several new or presumed new species were separately added from the Eastern Palaearctic and northern Oriental Regions by a long series of authors in the time span from 1845 to 1997, including two important (although very preliminary) revisions [31, 32] of both purported subgenera. Two Chinese species were added [33, 34], before the revision of the whole genus *Meligethes* [7], as currently delimited [2], and 23 new species (21 from China) have been described. Following this revision [7], a few other new species from China were also added [8–10], and other potential new species are presently under scrutiny based on recently collected Chinese material. The genus, comprising a new species still waiting for a formal description, but considered in the present analysis (*M.* sp. cfr. *Pectoralis* from S China; Table 1, and Tables 3–4 in Additional files), now includes 63 species in two subgenera (*Meligethes* s.str. and *Odonthogethes* Reitter, 1871) [7–10] (Table 1).

Most *Meligethes* (s.l.) diversity occurs in the southern countries of the Eastern Palearctic and in the northern portions of the Oriental Region (Middle Asia, China, Japan, N Indian subcontinent); but China certainly represents the main hot-spot of the genus, ca. 85% of the known species being at least marginally distributed in this country (Fig. 1 b–c, and Table 1) [7, 10].

**Fig. 2:**
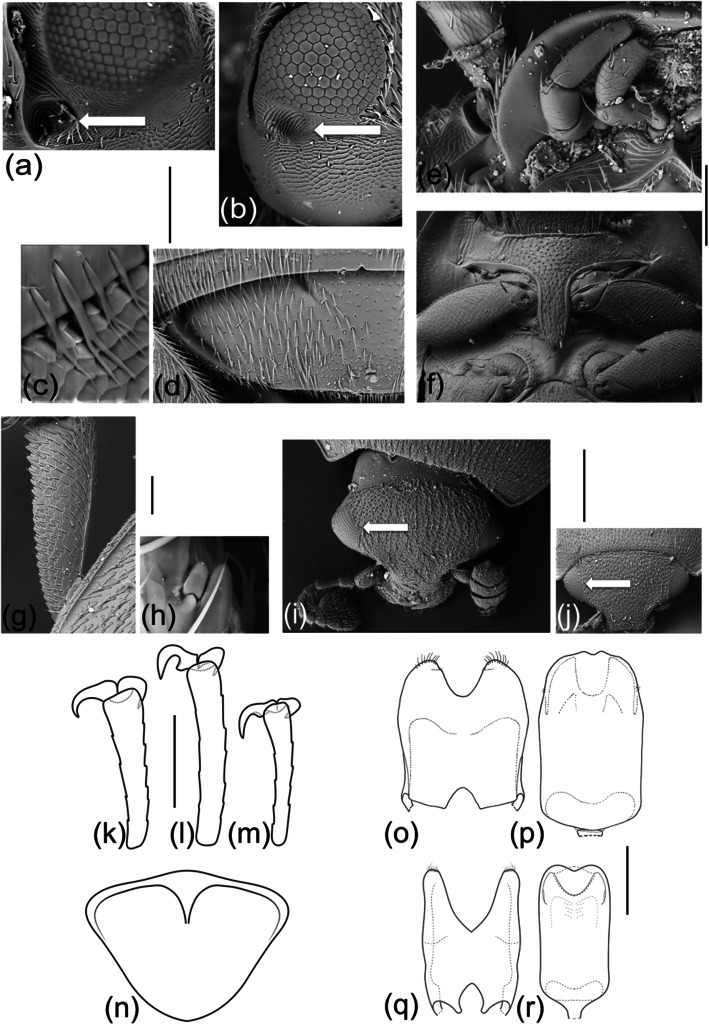
SEM pictures of *Meligethes* (s.l.) spp.; **a** left eye and ventral portion of temples (*Meligethes flavimanus*); **b** left eye and lateral portion of temples (*Odonthogethes castanescens*)*.* Arrows indicate shared pit in ventral subocular lateral portions of temples (inside terminal posterior portion of ventral furrows in members of subgenus *Meligethes* s. str., laterad and more dorsad in members of *Odonthogethes*). **c** Microsetae along posterior edge of pronotum of *Odonthogethes castanescens.* **d** Left portion of last visible abdominal ventrite of *Odonthogethes denticulatus.* **e** Right maxillary palp and labial palp of *Odonthogethes denticulatus.* **f** Prosternum, prosternal process and mesoventrite of *Odonthogethes denticulatus.* **g** Left protibia, dorsal view, of *Meligethes atratus*. **h** Close-up of left protibia, dorsal view, of *Odonthogethes castanescens,* with pre-distal tooth and spicule*.* Dorsal head view in Meligethinae; **i** *Meligethes atratus*; **j** *Brassicogethes aeneus.* Arrows of Figs (i) and (j) point to the shared absence of dorsal circumocular furrows on dorsal head surface. Drawings of *Meligethes* (s. l.) spp. (k-r); terminal left metatarsomeres: **k** *Meligethes atratus*; **l** *Odonthogethes denticulatus;* **m** *Odonthogethes flavicollis*. **n** Last visible dorsal abdominal segment (pygidium) of *Odonthogethes flavicollis*. Male genitalia (tegmen and median lobe of aedeagus) in *Meligethes* (s. l.) and *Brassicogethes*; **o**, **p** *Meligethes* (*M.*) *atratus*; **q**, **r** *Brassicogethes salvan* (Audisio, Antonini & De Biase, 2003)*.* Scale bar = 0.01 mm (Fig. h); = 0.02 mm (Fig. c); = 0.1 mm (Figs e, g, k-m); = 0.12 mm (Figs a, b); = 0.18 mm (Fig. d); = 0.2 mm (Figs o-r); = 0.4 mm (Figs i, j); = 0.5 mm (Figs f, n)

Species belonging to this supposed monophyletic genus are characterized by the following unequivocal autoapomorphic morphological and bionomical characters:

1) temples behind eyes (posterolateral view): with distinct, deep elliptical pit, positioned inside the posterior terminal portion of the antennal grooves (Fig. 2a); or with distinct, shallow, subcircular pit, placed more dorsad, outside antennal grooves, close to the posterior-lateral edge of the eye (Fig. 2b; pits on temples or inside antennal grooves are absent in all other Meligethinae exhibiting not raised notosternal sutures). 2) distal posterior portion of ventral antennal grooves (observed in ventral view): abruptly sloping, markedly delimited, deep, and distinctly wider than median portion (differently shaped in all other Meligethinae exhibiting not raised notosternal sutures). 3) larval development: on Rosaceae.

**Fig. 3:**
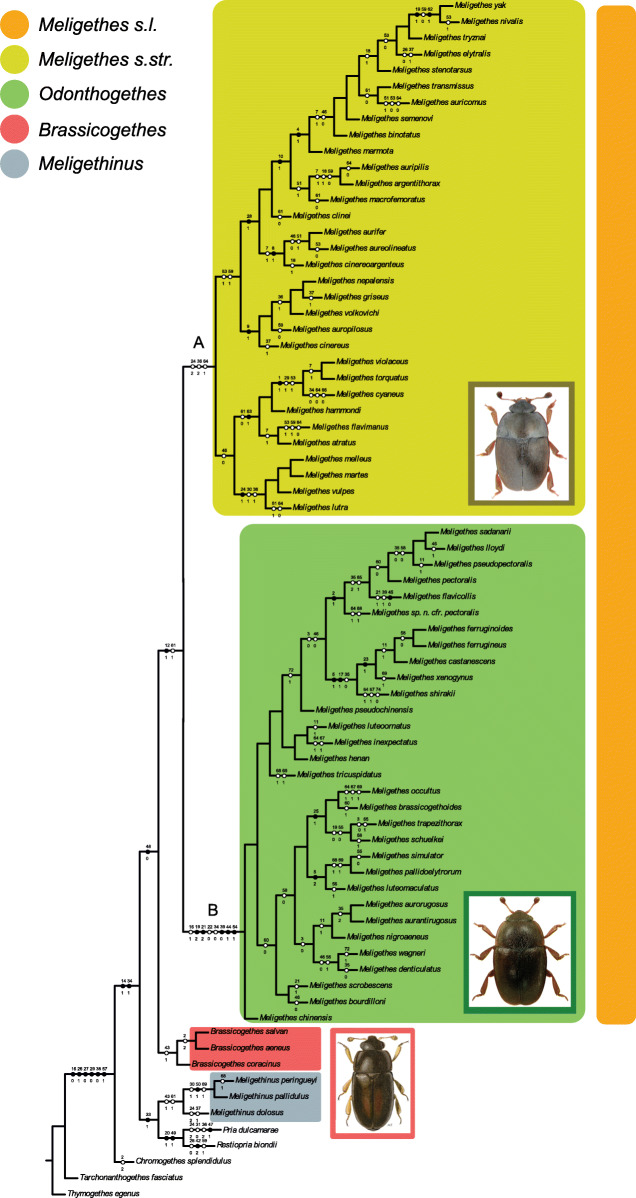
One of the most parsimonious trees based on morphological data (tree length = 184, C.I. = 0.45, R.I. = 0.87). Homoplasious and non-homoplasious character states are indicated with black and empty squares, respectively. Capital letters refer to clades as determined by morphological cladistic analysis (see Results and Discussion); the present-day subgeneric classification is superimposed on the C (*Odonthogethes*) and A + B (*Meligethes* s.str.) clades. Reconstruction is based on 74 morphological and ecological characters (169 states) for 63 members of *Meligethes, + Brassicogethes aeneus, B. salvan, B. coracinus, Meligethinus pallidulus, M. peringueyi, M. dolosus, Pria dulcamarae, Restiopria biondii, Tarchonanthogethes fasciatus, Chromogethes splendidulus,* and *Thymogethes egenus* (outgroups) (see Additional files Tables 3 and 4, for character list and matrix)

Several other morphological characters are shared with its supposed sister genus *Brassicogethes* Audisio & Cline, 2009 (Fig. 1, a (c)) [2, 5, 24, 26], whose included species all develop as larvae on the unrelated plant family Brassicaceae. These characters include, but are not limited to: 1) terminal tarsomere simple, not toothed at base (in all *Meligethes* s.str.; slightly to strongly toothed at base in *Odonthogethes*) (Fig. 2k-m); 2) terminal maxillary palpomera long and slender, ca. 3× longer than wide (Fig. 2e); 3) notosternal sutures usually not distinct, even in anterior portion (Fig. 2f), except in 3 species of *Meligethes* s.str.: *M. violaceus*, *M. torquatus* and *M. cyaneus* (Table 1 and Tables 3–4 in Additional files); 4) protibiae bearing only small, minute and subequal cuticular teeth along outer edge (Fig. 1 a (a), (b), (c), 2 g); 5) protibiae usually long and slender, up to 4–4.5× longer than wide (Fig. 1 a (a), (b), (c), 2 g); 6) pronotum scarcely convex, at least partially flattened at sides, with posterior angles almost right or (in most *Meligethes* s.str.) slightly turned posteriad (Fig. 1 a (a), (b), (c); 7) complete absence of circum-ocular furrows (“occipital sulci”) when viewed dorsally (Fig. 2 (i), (j)); 8) semi-circular arched impressions on both sides of the proximal basal portion of the last abdominal ventrite large and markedly distinct (Fig. 2 (d)); 9) semi-circular arched impressions on both sides of the proximal basal portion of the pygidium arcuately and regularly convergent distad (Fig. 2 (n)); 10) male genitalia with plesiotypic tegminal shape, tegmen characterized in most species by a deep, V-shaped incision (Fig. 2 (o)–(r)), similarly exhibited by several other basal Meligethinae genera, e.g., *Meligethinus* Grouvelle, *Micropria* Grouvelle, *Pria* Stephens, *Microporum* Waterhouse, and *Cryptarchopria* Jelínek.

### Trophic relationships of *Meligethes* with Rosaceae host-plants

Rosaceae is a middle-sized plant family that includes some 3000 species and a little less than 100 genera in 3 recognized subfamilies (Rosoideae, Spiraeoideae, and Dryadoideae) [35, 36]. Although exhibiting a worldwide distribution, Rosaceae are particularly diverse in northern Hemisphere temperate forests, where several genera and species of woody shrubs and small trees are important components of local forest communities. The family is peculiar in producing several different and highly distinctive types of fruits, including economically important edible fruits such as apples, pears, peaches, apricots, prunes, strawberries, cherries, raspberries, and blackberries. *Meligethes* s.l. (i.e., *Meligethes* s.str. + *Odonthogethes*) specialize on several genera and species of Rosaceae, although, differently from the majority of other Meligethinae lineages [2, 37, 38], only large shrubs and small trees of the two main subfamilies Rosoideae and Spiraeoideae are utilized [10]. No *Meligethes* (nor other Meligethinae genera) are, in fact, known to develop as larvae on herbaceous Rosaceae (e.g. the widespread and species-rich *Potentilla, Fragaria*, and *Geum*), despite species of these same plant genera being commonly used by adults of several different genera and species of Meligethinae as occasional food-plants [1].

Despite limited or incomplete information on the larval host-plants of some species, in general *Meligethes* s.l. appear to be associated with Rosaceae belonging to *Rosa*, *Rubus, Malus, Prunus, Crataegus, Pyracantha*, *Sorbaria*, and *Photynia* [1, 7, 10]. A couple of these (*Rosa*, *Rubus*) belong to the subfamily Rosoideae [35], whereas others (*Malus, Prunus, Crataegus, Pyracantha*, *Sorbaria*, and *Photynia*) to the subfamily Spiraeoideae. The two most commonly used larval hosts of *Meligethes* s.l. are *Rosa* L. and *Rubus* L. *Rosa*, which represents the only known larval host of species in the subgenus *Meligethes* s.str., includes some 200 species worldwide; *Rubus*, which represents the main larval hosts of species in the purported subgenus *Odonthogethes*, includes at least between 400 and 1000 species [35, 39–41]. Both genera exhibit biodiversity hot-spots in China and neighboring areas, where ca. 100 and more than 200 species respectively are known to occur [42]. A similar pattern occurs in *Meligethes* s.l., and in its constituent subgenera, species-groups and complexes [1, 2, 7]. Due to these above mentioned botanical phylogenetic and biogeographic scenarios, we comprehensively explored the evolutionary trajectories within *Meligethes* s.l. also to provide some hints on the role of the relationships between this group of pollen beetles and their larval host plants.

## Results

### Morphological phylogeny and cladistic analysis

The cladistic analysis of the matrix under implied weights (*k*10.72) yielded 429 equally most parsimonious trees with a total length of 184 steps, a consistency index (C.I.) of 0.45 and a retention index (R.I) of 0.87 (Fig. 3).

*Meligethes* s. l. and *Brassicogethes* were recovered as sister taxa as they shared 1 nonhomoplasious apomorphy (48:0, axillary triangular spaces close to proximal base at both sides of first abdominal ventrite larger than metatrochanter). The monophyly of *Meligethes* s.l. relied on 1 non homoplasious (12:1, postocular temples with pit) and 1 homoplasious (61:0) apomorphies. In turn, *Meligethes* included two main subclades: *Meligethes* s. str. (Clade A) and *Odonthogethes* (Clade B) (Fig. 3). *Meligethes* s. str. Was supported as monophyletic based on 3 homoplasious apomorphies (24:2; 36:2; 64:1). The monophyly of *Odonthogethes* was based on 5 nonhomoplasious (19:2, 3rd and 2nd antennomeres (ratio L03J/L02J) < = 0.9; 21:2, antennal club < 1.10–1.15 times as long as wide; 39:0, posterior edge of metaventrite outer posterior angles well distinct; 44:1, tarsal claws distinctly toothed at base; 54:1, longest setae on distal portion of parameres in dorsal view (ratio THLE/LETE) = > 0.14) and 3 homoplasious (16:1; 22:0; 34:0) apomorphies. The sister-group relationship between *Meligethes* s. l. and *Brassicogethes*, as well as the occurrence of the two main subclades within *Meligethes* s. l., i.e. *Meligethes* s. str. and *Odonthogethes*, was also confirmed by ML analysis of morphological data (Additional file 8).

### Molecular phylogeny and divergence time estimation

**Fig. 4:**
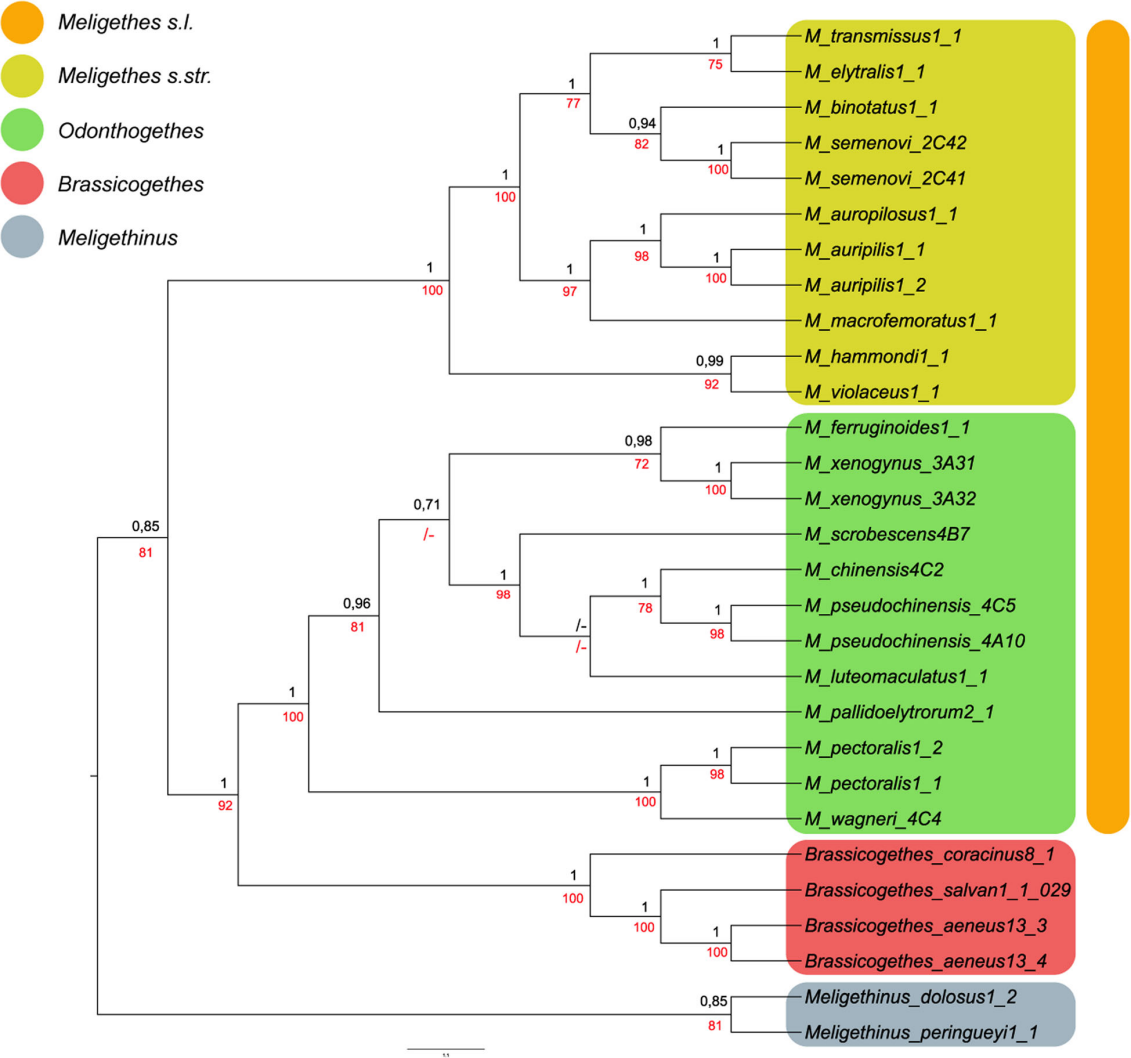
Phylogenetic interrelationships of representative members of *Meligethes* (s.l.) (*Meligethes* s.str. + *Odonthogethes*), *Brassicogethes,* and *Meligethinus* based on the concatenated molecular dataset (COI, 16S, CAD) using Bayesian inference (BI) performed by MrBayes, and maximum likelihood (ML) analyses performed by IQ-TREE. The final molecular data matrix includes 29 terminals and 1841 aligned characters. See Table 2, for details on the examined specimens. Only BI posterior probability (black) values and ML bootstrap (red) values exceeding 70% are shown

Our final dataset consisted of 1841 bp (COI: 578 bp, 16S: 491 bp, CAD: 772) obtained from 29 specimens (see Table 2). Phylogenetic trees resulting from the BI and ML analyses showed congruent topologies (Fig. 4); only BI posterior probability values and ML bootstrap values exceeding 70% are shown as BI/ML. Our phylogram indicated the presence of two highly supported principal clades, corresponding to the *Meligethes* s.str. Species (BI = 1/ML = 100) and two clades including *Odonthogethes* and *Brassicogethes* species, respectively (BI = 1/ML = 92). Moreover, the monophyly and the sister-group relationships of a clade including *Meligethes* s.str. + [*Odonthogethes* + *Brassicogethes*] was well supported in both BI (0.85) and ML (81). The trees obtained from single-gene alignments are reported in Additional files (4, 5 and 6).

**Fig. 5:**
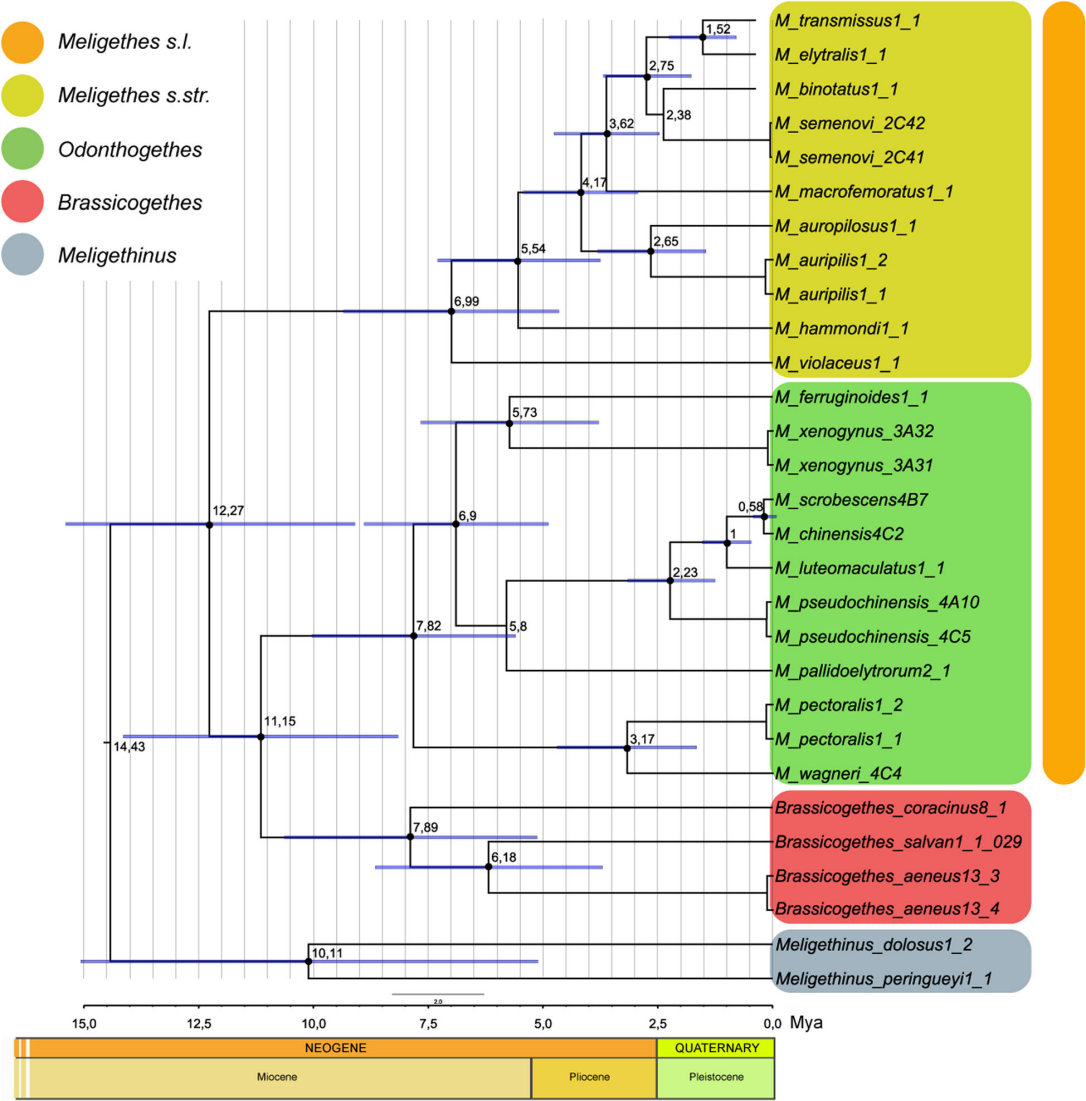
Time-calibrated BEAST phylogeny of representative members of *Meligethes* s.str., *Odonthogethes*, *Brassicogethes* and *Meligethinus*, inferred from combined mitochondrial sequences (COI, 16S). Numbers at nodes correspond to estimated age (Mya) obtained with calibration of 0.0126 substitutions/site per My; bars represent highest posterior densities (95%) around mean date estimates. Nodes with black dots were supported with high posterior support (> 95)

Divergence time estimates from BEAST are depicted in Fig. 5. With a calibration of 0.0126 substitutions/site per My, the possible origin of the core members of the *Meligethes* complex of genera [*Meligethes* s.str. + *Odonthogethes* + *Brassicogethes*] (i.e., divergence from *Meligethinus*) was estimated to be approximately in the Middle Miocene, ca. 15–14 Mya. The split of *Odonthogethes* from *Meligethes* can be traced back to the Middle Miocene (12.27 Mya; 95% HPD: 9.10–15.30 Mya) and that of *Brassicogethes* from *Odonthogethes* in the Late Miocene (11.15 Mya; 95% HPD: 8.20–14.10 Mya).

### Molecular vs. morphological analyses

Evidence from our molecular analyses resulted in a rather good agreement with previously established morphology-based systematics of *Meligethes* and allied genera. Our molecular analyses suggested (Figs. 4-5) that most *Odonthogethes* species likely differentiated within the last 10 million years. These were mostly included in the *O. chinensis* species-group, the largest one within this genus, which are associated as larvae with *Rubus* spp. (Rosoideae) and with several other Spiraeoideae. Although the taxon sampling in our molecular analysis is rather partial, a preliminary conclusions and comparison with morphological-based results, allows confirming the monophyly of the present-day *Odonthogethes*. Nevertheless, the monophyly of the (sub) genus *Meligethes* s. str. Remains slightly ambiguous morphologically, suggesting that this taxon might be an assemblage from which *Odonthogethes* was derived. A future work with additional material of representatives of the *M. vulpes* species-group (Fig. 3; *Meligethes vulpes* and allied species, mostly from hardly accessible countries in Middle Asia) is needed to verify, through molecular data, the phylogenetic position of this isolated group within *Meligethes* s. str. Additionally, our molecular analysis recovered *Brassicogethes* as deeply nested within *Meligethes* pointing to a paraphyletic condition of the genus *Meligethes*, as recently conceived. This evidence is partially in contrast with morphological analyses, which, on the contrary, suggests a sister-group relationship between *Brassicogethes* and (a monophyletic) *Meligethes* [*Meligethes* s.str. + *Odonthogethes*] (the tree obtained from combined molecular and morphological dataset is reported in Additional file 7). In presence of this contrasting information, the hypotheses to either downgrade *Brassicogethes* to the subgenus level, within the genus *Meligethes* s. l., or, alternatively, to raise *Odonthogethes* to the genus rank should be taken into account.

### Ancestral state parsimony reconstruction of larval-host-plant associations

**Fig. 6:**
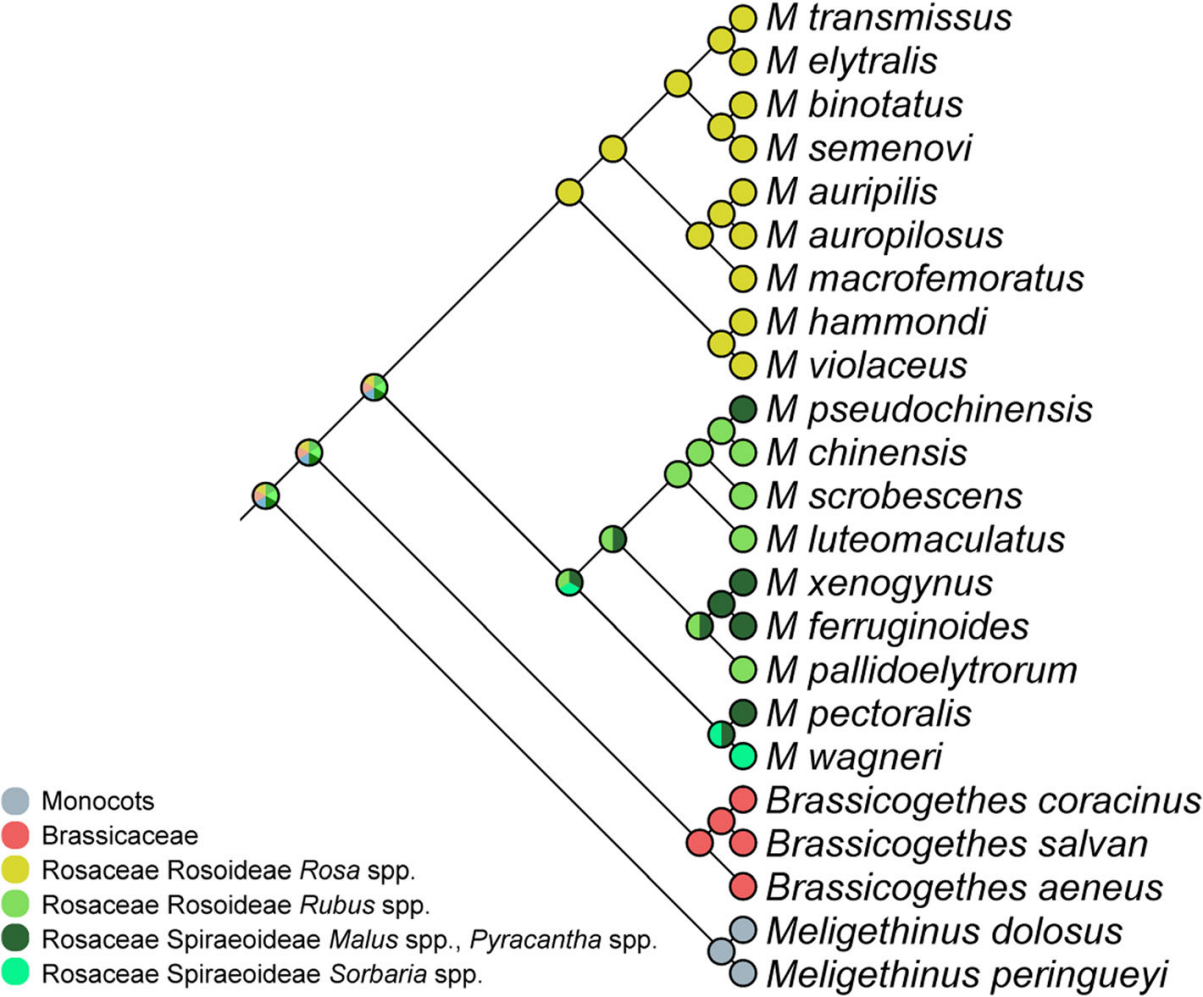
The strict consensus tree based on combined morphological and molecular dataset constructed via MrBayes, superimposed on ancestral state parsimony reconstruction of larval-host-plant associations executed in MESQUITE (tribes within Rosaceae according to [35, 36, 39–41])

The ancestral state reconstruction plotted on the topology resulting from the combined morphological and molecular dataset analysis (Fig. 6) suggested that Rosaceae could be regarded as the ancestral host-plant family of *Meligethes* (s.l.) and *Brassicogethes,* although the actual succession of family-level larval shifts among the whole clade remains difficult to define.

Noteworthy, *Meligethinus* species are all associated with monocots in the family Arecaceae [1, 2, 43]. According to our molecular data, the common ancestor of *Meligethes* s. l. subsequently colonized a new niche, probably exploiting Rosaceae in the Eastern Palaearctic, while *Brassicogethes,* maybe also originated in the same areas, later radiated on Brassicaceae, mostly in W Palaearctic subregion. This scenario is supported by the ancestral state reconstruction, which suggested that the diverse genus *Rosa* L. most likely represented the ancestral host of *Meligethes* s. str. The larvae of most species of *Meligethes* s. str. Specialized on species belonging to this plant group, while members of *Odonthogethes* rather shifted on other Rosoideae, specifically on the genus *Rubus* L. Subsequently, a species of this subgenus probably colonized Rosaceae belonging to the subfamily Spiraeoideae and this ancestral host shift event may have led to a subsequent diversification of a group whose members radiated by exploiting several plants of this subfamily (e.g. *Malus*, *Crataegus*, *Pyracantha* and *Photinia*) (Fig. 6). A few isolated species belonging to both subgenera, *Meligethes* (*M. atratus*, *M. flavimanus*, and *M. torquatus*) and *Odonthogethes* (*O. shirakii*) also independently colonized Spiraeoideae of the genus *Prunus* [1, 13]. An even more isolated host shift event was likely experienced by *Odonthogethes wagneri*, the only species feeding on Spiraeoideae of the genus *Sorbaria*, and the isolated *O. flavicollis*, which is the only species apparently feeding on Spiraeoideae of the genus *Photinia* [10]*.* Molecular data suggest that a more drastic ecological shift finally involved some ancestral species of *Odonthogethes,* able to transfer to Brassicaceae, and very likely originating the *Brassicogethes* clade.

## Discussion

*Meligethes* originated in the SE Palaearctic and NE Asia and adapted to flowers and large inflorescences of Rosaceae for larval feeding. The genus was likely derived from an ancient stem of Meligethinae not distantly related to *Meligethinus*, *Micropria*, *Kabakovia, Pria*, and allied genera, several species of which (except among *Pria* and *Micropria*) are associated as larvae with male inflorescences of palms (Arecaceae) [1, 2, 24, 26, 43].

As for the timing of lineage splitting, the few fossil data available for Meligethinae [44, 45] are based on Baltic amber material (traditionally dated back to some 36–38 Mya). However, although certainly to be referred to genera other than *Meligethes* as presently circumscribed, the correct classification of these fossils is still pending. The Baltic amber material from Oligocene (ca. 40–50 Mya; “*Melipriopsis rasnitzyni*”) [45], is, for instance, hardly referable to Meligethinae, due to some characters (e.g. the distinctly bordered posterior base of pronotum; the angulose “axyllary line” on metaventrite; the ciliate pronotal sides) which are unknown among true members of this subfamily. Due to the above-mentioned uncertainties, we were not able to rely on distant external calibration points [46]. It is worth remarking, however, that even if distantly related fossils were available; these would have improperly timed the evolution of the focal group due to its intrinsic biological properties [47]. In fact, Meligethinae are known to be affected by rate variation among lineages, incomplete haplotype sorting and a host-plant dependent and fluctuating demography [22, 23]. Hence, we decided to tentatively provide a temporal framework for the speciation events of the target taxa by relying on the most accurate substitution rates currently available for beetles and setting a relaxed clock model to overcome some of the recognized limits of this dating method [46, 47]. Our molecular analyses suggest that timing of separation between [*Meligethes +* [*Odonthogethes* + *Brassicogethes*]] occurred in a time frame of ca. 11–13 Mya, whereas separation of this clade from the related genus *Meligethinus* probably dates back to 14–15 Mya. Therefore, the Middle Miocene (Langhian Age) could represent a plausible timing for the first *Meligethes* lineages to specialize on Rosaceae host plants. *Meligethes* later continued to evolve and adapt in Eastern Palaearctic and E Asiatic areas on Rosaceae Rosoideae, from which *Odonthogethes* plausibly radiated during the Late Miocene, Pliocene and Pleistocene on Spiraeoideae (Rosaceae subfamily). *Brassicogethes,* following their shift to Brassicaceae - likely from a stem of ancestral *Odonthogethes* - expanded and strongly diversified its constituent lineages mostly in W Palaearctic, paralleling a westward evolution, diversification and expansive radiation of Brassicaceae host-plants. Several closely related species of this (sub) genus likely differentiated only in the last 2 Mys [14, 22, 23]. These species, in fact, usually exhibit low levels of molecular interspecific differentiation, typical of recently specialized lineages among which genetic differentiation can be markedly slower than diversification among morphological and ecological adaptive traits. This phenomenon, well-known in different groups of recently speciated phytophagous insects [23], is probably due to the retention of common ancestral haplotypes when genetic differentiation has not had the necessary time to be fixed after speciation [23]. Among *Odonthogethes*, a similar phenomenon certainly involves most members of the *Odonthogethes chinensis* group, which, although strongly differentiated from one another based on morphological and ecological traits, exhibit low levels of interspecific genetic differentiation (Fig. 5) [Audisio et al. unpublished data].

Only a few *Meligethes* of the (sub) genera *Meligethes* and *Odonthogethes* were able to reach the W Palaearctic, likely during the most recent Pleistocene Glaciation Cycles, maybe due to the moderate number of potential larval host-plants (indigenous Rosaceae) occurring west of Middle Asia. Mountain systems of the SE Palaearctic (in particular those in Central and S China, Nepal, Bhutan, and NE India) and transitional areas between E Palaearctic and N Oriental Regions, seem to represent the most active centers of speciation and evolution of *Meligethes* and *Odonthogethes*, reflecting the local strong generic- and species-level diversification of Rosaceae [35, 36, 40–42].

As mentioned above, *Meligethes* s. str. Appear to be more strictly specialized on *Rosa* spp., while *Odonthogethes* appear to have adapted to a much wider range of Rosaceae genera, including: *Rubus* to *Prunus*, *Sorbaria* and related taxa, in the two main Rosaceae subfamilies, Rosoideae and Spiraeoideae [35, 36, 48]. The only known more highly polyphagous *Meligethes* s.str. Are represented by two species (*M. atratus* and *M. flavimanus)* that are widespread from N Asia to the Iberian Peninsula. Both species are able to develop, at least in W Palaearctic, on different genera of Rosoideae and Spiraeoideae [1, 7]. This could be interpreted as a recent local widening of ecological (host-plant) ranges, following post-glacial colonization of W Europe, in a region where other competing and more specialized Rosaceae-dependent species of the same genus are absent.

The two largest genera of Rosaceae Rosoideae, *Rubus* and *Rosa*, split more than 40 Mya, well before the here estimated divergence between *Odonthogethes* (mostly associated with *Rubus* spp.) and *Meligethes* s. str. (all likely associated with *Rosa* spp.) that is tentatively dated ca. 12–13 Mya. This asynchronous timing clearly does not allow supporting the hypothesis of a direct coevolution [49] among these beetles and their host plants. However, a model of ‘sequential evolution’ - i.e. the shift of insect herbivores onto a pre-existing group of plant species [50] - could rather be raised to explain the origin of the main (deepest) specialized-clades by some ancient common ancestors able to colonize novel plant-groups of Rosoideae (Figs. 5-6). Yet, it is worth noticing that most strictly endemic and locally distributed *Meligethes* and *Odonthogethes* species from China and neighboring countries are typically associated as larvae with common and widespread floral hosts, and not to endemic plant species [10]. Therefore, geographic isolation in more or less remote mountain valleys seems to have played a more relevant role than ecological specialization to host plants in driving speciation among members included in both *Meligethes* and *Odonthogethes* clades. It is interesting to point out that *Meligethes* and *Odonthogethes* probably experienced different selective pressures respect to the W Palaearctic *Brassicogethes* (associated with the unrelated family Brassicaceae), whose more recently-speciated taxa were able to frequently colonize and adapt to rare and/or endemic plant species [1, 5, 14–23].

In line with the above argument, we observed that morphology allowed to set apart and identify the main lineages of both *Meligethes* s.str. and *Odonthogethes*, although the extensive homoplasy between these groups prevented to fully resolve the phylogenetic relationships among species-groups (Fig. 3, Additional file 8). Additionally, several characters of both taxa, although diagnostic at the interspecific level, were widely affected by homoplasy. The sharing of so many morphological traits could suggest that members of several species-group in *Meligethes* s.str. and *Odonthogethes*, probably originated recently (e.g., in allopatry) without experiencing the strong selective pressures posed by the adaptation to numerous larval host plants and various ecological contexts, which, on the contrary, rather favored a (relatively) more rapid diversification of *Brassicogethes*. Consistently, in *Brassicogethes*, where combined phenomena of speciation triggered by larval host-plant specialization (following repeated larval host-shifts) occurred much more frequently, phylogenetic relationships among the main species-groups appear to be more clearly resolved by morphological data alone, though relationships within some morphologically recognized species groups required the support of molecular data as well [23].

We are confident that further analyses could provide more detailed phylogenetic evidence on the three involved lineages, in a more coherent scenario of their actual evolutionary relationships, despite currently partly contrasting phylogenetic information provided by morphological data and molecular markers. Additionally, molecular data suggests a markedly isolated phylogenetic position for *Meligethinus dolosus* Grouvelle, 1919 (from NE South Africa and Mozambique). This evidence is confirmed by morphological data [43]. This problem, as well as the internal phylogeny of *Meligethinus*, is beyond the scope of the present paper, and will be discussed in an upcoming effort that is specifically devoted to a morphological and molecular phylogeny of this genus [43].

## Conclusions

The present study analysed 63 species of *Meligethes* (s.l.), setting up the basis for further research on this taxon. Judging from the relatively high number of new species (> 12) discovered during less than five years of field and Museum research following the recent revision of this group [7], combined with the vast extension of scarcely explored E Palaearctic and N Asian areas (eastern Middle Asia, Northern Indian subcontinent, S China, and northern Indochina), there is a high likelihood that the actual number of species in the (sub) genera *Meligethes* and *Odonthogethes* might increase significantly to even more than 70–80 species as a whole. The species of both clades seem to be concentrated in central and southern China, specifically in subtropical evergreen broadleaf forest zones, in eastern portions of the Qinghai-Xizang Plateau alpine vegetation zone, and in southern portions of the warm temperate deciduous-broadleaf forest zone [51] (http://www.chinamaps.org/china/china-land-cover-map-large-2.html). However, scarcely explored mountain areas of S China still possess new endemic species. These areas represent true biodiversity hot-spots (Fig. 1 b–c) for both *Meligethes* and *Odonthogethes* and their Rosaceae host-plants, and also likely include Nepal, Bhutan, Taiwan, and the nearly unexplored surrounding mountain areas of eastern India (Arunachal Pradesh) and northern Myanmar [7–10].

Despite the incomplete taxonomic and biogeographic coverage, the present morpho-ecological phylogenetic analysis and the thus far available molecular data seem to support at least the following conclusions:

1) Based on molecular evidence, the *Meligethes* complex of genera univocally represents a monophyletic lineage, including almost certainly three species-rich genera or subgenera (*Meligethes* s.str., *Odonthogethes,* and *Brassicogethes*), which are comparable in the number of inclusive species (32, 31 and 42 species, respectively). Other preliminary molecular data using mitochondrial and nuclear markers (COI, NADH, ITS2, PEPCK) [24, 26], suggested that the genus *Meligethinus* Grouvelle (a small, mostly Oriental and Afrotropical genus including some 20 species associated as larvae with male inflorescences of Arecaceae [1, 43]) is placed as a sister taxon of this clade. In fact, the phylogenetic position of *Meligethes* and *Odonthogethes* is certainly closer to both *Brassicogethes* and *Meligethinus*, than to any other recognized genera inside Meligethinae [43]. Indeed, the positional homologous insert at the apical portion of the *ITS2* domain B, which, as previously demonstrated [24], is shared across all *Meligethes, Odonthogethes, Brassicogethes* and *Meligethinus*, should be considered a diagnostic sequence insertion. This combined with other slippage-derived signature sequences identified in the same paper for these related Meligethinae genera strongly corroborates a common origin of these four taxa.

2) Based only on morphological and ecological evidence (Fig. 3), the clade [*Meligethes* s.str. + *Odonthogethes*] could represent the sister-group of the genus *Brassicogethes*, with *Meligethinus* sister of this triplet of taxa. However, the molecular data presented here (Figs. 4-5) strongly suggest a different and likely more compelling scenario, with *Brassicogethes* and *Odonthogethes* being sister taxa, *Meligethes* being sister of the latter clade, and *Meligethinus* sister of this triplet of taxa.

3) In an ecological-evolutionary perspective, differently from *Brassicogethes*, geographic isolation in remote mountain valleys of the Eastern Palaearctic seems to have played a more relevant role than ecological specialization in species splitting and diversification within both *Meligethes* and *Odonthogethes* genera.

Finally, a short series of very recently published or upcoming papers of our research team is aimed to introduce and formalize an updated taxonomic rank for *Meligethes, Odonthogethes*, and *Brassicogethes*, and to describe some recently discovered new Chinese species, as well as the previously unknown males of a few additional taxa [52].

## Methods

### Field research

All species were collected as adults on their known or putative host-plants. Attempts were also made to obtain larvae to confirm the insect-host plant relationships. Larvae were collected alive by hand using entomological forceps inside flower buds, where they mainly develop during the early flowering season of hosts. Adults destined for morphological analyses were killed in small vials containing cork powder and a few drops of ethylacetate. Additional conspecific adults destined to molecular analyses were killed and preserved in absolute ethanol. Larvae destined for morphological analyses were killed and preserved in a mixture of 75% ethanol and 25% filtered pure water, while additional conspecific specimens destined for molecular analyses were killed and preserved in absolute ethanol. A few larvae, when available in number, destined to SEM analyses, were killed in absolute acetone to preserve the larval cuticular surface and corresponding setae, tubercles, and other projections.

In most cases, adult specimens were collected after direct observations on target plants, with some specimens of possible target beetles directly collected using an aspirator or by hand. This technique had to be employed to avoid physical damage to rare and locally protected plants via netting or beating techniques. Netting or beating were used only for locally abundant host-plants, such as widespread or even invasive species of the *Rubus* or *Prunus* in shrubby habitats. Pollen beetles on these plants are usually highly dispersed and therefore difficult to find and collect using visual observation only.

### Morphological phylogeny and host plant associations

A matrix containing 69 morphological and 5 ecological characters (Tables 3–4) was compiled for all known 63 species of *Meligethes*. The genera *Thymogethes* Audisio and Cline, 2009 (represented here by *T. egenus* from S Europe), *Brassicogethes* Audisio and Cline, 2009 (*B. aeneus, B., salvan* and *B. coracinus* from S Europe), *Tarchonanthogethes* Audisio and Cline, 2009 (*T. fasciatus* from S Africa), *Chromogethes* Kirejtshuk, 1989 (*C. splendidulus* from S Africa), *Restiopria* Audisio, Jelínek and Cline, 2011 in [25] (*R. biondii* from S Africa), *Pria* Stephens 1830 (*P. dulcamarae* from S Europe) and *Meligethinus* Grouvelle, 1906 (*M. pallidulus* from W Mediterranean areas, and *M. peringueyi* and *M. dolosus* from Southern Africa) were selected as outgroups based on their different levels of affinities with *Meligethes* Stephens [5, 6, 18]. Among the 69 selected morpho-ecological characters, some usually significant in Meligethinae interspecific diagnostics (e.g., some from male and female genitalia) were not included. Those traits, upon cladistics analysis, resulted manifestly homoplasious in different unrelated clades, and therefore were deemed scarcely informative or even confusing in a cladistic framework. The morphological matrix was produced with MESQUITE version 3.51 [52] and subsequently analyzed in TNT version 1.5 [53]. Multistate characters were treated as unordered and zero-length branches were collapsed. Analyses were run as implicit enumeration under implied weights (concavity factor of 1 and higher), under the “traditional search option”. The following parameters were applied: general RAM of 1 GB, memory set to hold 1,000,000 trees, setting 1000 replicates with tree bisection-reconnection branch swapping and saving 1000 trees per replicate. The most fitting concavity *k*-value of the weighting function was found using the TNT script “setk.run” [53], obtaining a *k* value 10.72. Characters were mapped on one of the most parsimonious trees using Winclada version 1.00.08 [54]. To confirm the deepest relationships inferred by the MP analysis, we also generated a Maximum Likelihood (ML) tree using default settings in IQ-TREE [55].

An ancestral state parsimony reconstruction of larval-host-plant associations was carried out in MESQUITE version 3.51 [56] using the most recent phylogeny-based general classification schemes of Rosaceae [35, 36]. The reconstruction of ancestral state parsimony for larval-host-plant association (Fig. 6) was carried out and superimposed on our combined morphological and molecular dataset-based cladogram following the methods discussed elsewhere [23].

### Insect-host plant relationships and phenology

We collected field information on life history, larval host-plants and phenology on ca. 45 out of 63 species of the genus; for some poorly known and rare species from N Indian subcontinent, Middle Asia, China and Japan no additional information on biology was provided. Among the analyzed species, steno-oligophagy (i.e. dependence of a single beetle species on a short series of closely related plant species belonging to the same genus or to closely related genera) is the dominant condition, while monophagous species are few.

Most species of the genus are active during the breeding season in Summer (i.e., mid-June to late July; 36 species out of 45, i.e., 80%), while only a couple of mostly W Palaearctic species are Spring specialists (breeding from early April to middle May; 2 species out of 45, i.e., 9%: *M. atratus* and *M. flavimanus*). The remaining 7 analyzed species more widely extend their annual phenology between May and August.

### Molecular methods

#### DNA extraction, amplification and sequencing

A total of 29 adult specimens from 18 different *Meligethes* s.l. species*,* as well as 3 species of *Brassicogethes* and 2 of *Meligethinus* were collected alive in the field and directly killed and preserved in absolute ethanol. In Table 2, the geographic details for the species are listed. Species identifications were made using morphological characters detailed elsewhere [1, 7–9] and in Table 1 and Table 3 in Additional files. Total genomic DNA was extracted from whole specimens, following the salting out protocol [57]. Sequences were obtained from two mitochondrial gene fragments, the Cytochrome Oxidase subunit I (COI) and 16S rRNA (16S); and from one nuclear fragment, a portion of the rudimentary gene (CAD). For PCR amplifications, the following primer pairs were used: COI = LC01490 5′-.

TCAACAAATCATAAAGATATTGG-3′ HC02198 5′-TAAACTTCAGGGTGACCAA AAAATCA-3′ [58]; 16S = 16SA5’-CGCCTGTTTATCAAAAACAT- 3′; 16SB 5′- CTCCGGTTTGAACTCAGATCA- 3′ [59]; CAD = CD439F 5′-TTCAGTGTACARTTYCAYCCHGARCAYAC-3′ CD688R 5′-TGTATACCTAGAGGATCDACRTTYTCCATRTTRCA-3′ [60].

Amplifications of the mitochondrial genes were performed with the following general cycle conditions: initial denaturation at 96 °C for three minutes, followed by 35 cycles of denaturation at 94 °C for one minute, annealing at 54°-57 °C for 40 s, 1-min. Extension at 72 °C and a last 7-min. Elongation step at 72 °C. Reactions were performed in a 25 μl volume containing (NH_4_)_2_SO_4_ 16 mM, Tris–HCl 67 mM (pH 8.8 at 25 °C), MgCl_2_ 3 mM, 1 mM of each dNTP, 0.8 pmol of each primer and 1.25 units of Taq DNA polymerase. A touchdown PCR protocol was used to amplify the CAD marker with the following thermal cycling conditions: 94 °C for 3.5 min, followed by 20 cycles of 94 °C, 30 s, annealing temperatures stepdowns every cycle of 0.4 °C (from 58 to 50 °C), 35 s, 72 °C, 2.5 min and additional 20 cycles of 94 °C, 30 s, 55 °C, 35 s, 72 °C, 2.5 min. We used an MJ MINI Personal Thermal Cycler (BIO-RAD Laboratories, US) and LifeECO Thermal Cycler to perform PCR amplifications. The PCR products were purified with a GENEAID- Gel/PCR DNA Fragments Extraction Kit and sent to an external sequencing service (Macrogen Inc.: www.macrogen.com). Sequences were edited and aligned with GENEIUS v9.1.6 [61]. A total of 84 new sequences have been deposited in GenBank (Accession numbers COI: MT949495-MT949523; 16S: MT957149-MT957177; CAD: MT966846-MT966871).

#### Molecular phylogeny and divergence time estimation

Phylogenetic analyses (BI) were first performed using single-gene alignments and then, Bayesian inference (BI) and Maximum likelihood analysis (ML) were both performed on the concatenated (mtDNA + nucDNA) dataset using, respectively, MRBAYES v3.2.1 [62] and IQ-TREE [55] as implemented in W-IQ-TREE [63]. The best-fit models for the study data sets proved to be a Generalized Time- Reversible model with a proportion of invariable sites and heterogeneous substitution rates following a gamma distribution (GTR + I + G) for the COI and 16S and Generalised Time-Reversible model with a proportion of invariant sites (GTR + I) for CAD. The best fitting model to analyse each partition was selected by JMODELTEST [64] using the Akaike information criterion. The BI analysis was performed by running 5,000,000 generations, with Markov chains sampled every 1000 generations. A 10% burn-in was applied and the remaining trees were used to compute a 50% majority rule consensus tree and posterior probabilities. We assessed convergence of the runs by investigating the average standard deviation of split frequencies, potential scale reduction factor (PSRF) in MRBAYES and effective sample size (ESS) of all parameters in TRACER 1.6 [65]. Average standard deviation below 0.01, value of PSRF close to 1.00 and ESS values over 200 for all parameters were acknowledged as good indicators of convergence. A ML phylogenetic reconstruction was performed running 1000 ultrafast bootstrap replications [66] followed by 1000 replications of assessment of branch supports with single branch tests with SH-like approximate likelihood ratio test. The best fitting model to analyze each partition was selected as for BI.

To estimate the relative age of lineage divergences, an uncorrelated lognormal Bayesian molecular relaxed clock model and a Yule process prior were used on the mtDNA data set using the software BEAST v.1.8.2 [67]. Although some fossils ascribed to Meligethinae have been described and dated from Baltic ambers [44, 45], these oldest representatives require a careful revision by other specialists of this group, which consider them either doubtfully attributable to *Meligethes* or even to the same Meligethinae subfamily. Hence, given the lack of reliable fossil records or useful dated palaeogeographic events to calibrate our trees, we used an average value of the COI substitution rate in a range between 1.5 and 3.54%, which represent the most accurate estimated rate for mitochondrial DNA in coleopterans so far [68–70] and hence widely applied in molecular dating of beetle phylogeography and phylogeny (e.g. 6, 23, [71–75]). Therefore, for the molecular clock analysis we applied an average rate of 0.0126. The GTR model was transferred to the HKY [76, 77] model because there are low ESS values for some parameters in the analyses when applying the GTR model. The analysis was independently performed three times, with 100 million generations and sampling of trees every 10,000 steps. Effective Sample Size (ESS) was evaluated in Tracer v1.6 [65], considering runs with ESS values above 200. Output trees were generated in Tree Annotator v1.8.2 (BEAST package), using maximum clade credibility (MCC) after a 10% burn-in and median heights.

## Supplementary Information

**Additional file 1.** Abbreviations. (Museum acronyms following Evenhuis NL. The Insect and Spider Collections of the World Website. 2020http://hbs.bishopmuseum.org/codens/ [accessed at March 26th, 2020]).

**Additional file 2 **Table 3 List of morphological and bionomical characters used in the cladistic analysis on all 63 *Meligethes* s.l. [*Meligethes* s.str. + *Odonthogethes*] species and on selected outgroup species, including *Brassicogethes*.

**Additional file 3 **Table 4 Matrix of the character states in the 63 *Meligethes* s.l. species and in the selected purported 7 outgroup species and genera, including *Brassicogethes*.

**Additional file 4 **The Bayesian tree of *Meligethes*-complex on analyses of the mitochondrial COI gene. The posterior probabilities exceeding 50% are shown at nodes.

**Additional file 5 **The Bayesian tree of *Meligethes*-complex on analyses of the mitochondrial 16S gene. The posterior probabilities exceeding 50% are shown at nodes.

**Additional file 6 **The Bayesian tree of *Meligethes*-complex on analyses of the nuclear CAD gene. The posterior probabilities exceeding 50% are shown at nodes.

**Additional file 7 **The Bayesian tree of *Meligethes*-complex based on combined molecular and morphological dataset. The posterior probabilities exceeding 50% are shown at nodes.

**Additional file 8 **The Maximum Likelihood (ML) tree of *Meligethes*-complex based on morphological data, performed by IQ-TREE using default settings.

## Data Availability

Sequence data (mtDNA and nDNA) can be found in GenBank (see Table 2). Material of Meligethinae studied for the aims of the present research was examined from or is preserved in the institutions and private collections listed above under Abbreviations.
