# Supplementary material for: Rosaceae, Brassicaceae and pollen beetles: exploring relationships and evolution in an anthophilous beetle lineage (Nitidulidae, Meligethes-complex of genera) using an integrative approach
Source: Front Zool. 2021 Mar 6;18:9. doi: 10.1186/s12983-021-00390-4 (PMC7936458; doi:10.1186/s12983-021-00390-4)
Supplement: Supplementary file 1 — Additional file 1. Abbreviations. (Museum acronyms following Evenhuis NL. The Insect and Spider Collections of the World Website. 2020http://hbs.bishopmuseum.org/codens/ [accessed at March 26th, 2020]). [file 12983_2021_390_MOESM1_ESM.docx]

**Abbreviations**

(Museum acronyms following Evenhuis NL. The Insect and Spider Collections of the World

Website. 2020; http://hbs.bishopmuseum.org/codens/ [accessed at March 26th, 2020])

ARCC – A.R. Cline’s collection, housed at the California State Collection of Arthropods in

Sacramento, California, USA

BMNH – Natural History Museum, London

CAL – A. Lasoń’s collection, Bialystok, Poland

CAR – P. Audisio’s collection, currently housed in the Zoological Museum, Sapienza Rome

University, Rome, Italy

CAS – California Academy of Sciences, Sacramento, USA

CHHU – Sadatomo and Sadanari Hisamatsu’s Collection, The United Graduate School of

Agricultural Sciences, Ehime University, Japan

CSCA – California State Collection of Arthropods,Sacramento, California, USA

ETHZ – Eidgenössische Technische Hochschule, Entomologisches Institut, Zürich, Switzerland

IZAS – Institute of Zoology, Chinese Academy of Sciences, Beijing, China

HNHM – Hungarian Natural History Museum, Budapest, Hungary

MAKB – Zoologische Forschungsinstitut und Museum “Alexander Koenig”, Bonn, Germany

MHNG – Muséum d’Histoire Naturelle, Genève, Switzerland

MNHN – Muséum National d´Histoire naturelle, Paris, France

MNST – National Museum of Natural Science, Taichung, Taiwan NHMB – Naturhistorisches

Museum, Basel, Switzerland

NHMW – Naturhistorisches Museum, Wien, Austria

NKME – Museum für Naturkunde, Erfurt, Germany

NKMS – Museum für Naturkunde, Stuttgart, Germany

NMPC – National Museum, Prague, Czech Republic

PANW – Institute of Zoology, Polish Academy of Sciences, Warszawa, Poland

RSC – R. Schuh collection, Wiener Neustadt, Austria

SMF – Forschungsinstitut und Naturmuseum Senckenberg, Frankfurt-am-Main, Germany

ZIN – Zoological Institute, Academy of Sciences, St. Petersburg, Russia

ZMUM – Zoological Museum, Moscow State University, Moscow, Russia

ZSM – Zoologische Staatssammlung, München, Germany
