# Supplementary material for: Rosaceae, Brassicaceae and pollen beetles: exploring relationships and evolution in an anthophilous beetle lineage (Nitidulidae, Meligethes-complex of genera) using an integrative approach
Source: Front Zool. 2021 Mar 6;18:9. doi: 10.1186/s12983-021-00390-4 (PMC7936458; doi:10.1186/s12983-021-00390-4)
Supplement: Supplementary file 2 — Additional file 2 Table 3 List of morphological and bionomical characters used in the cladistic analysis on all 63 Meligethes s.l. [Meligethes s.str. + Odonthogethes] species and on selected outgroup species, including Brassicogethes. [file 12983_2021_390_MOESM2_ESM.docx]

| **Table 3** List of morphological and bionomical characters used in the cladistic analysis on all 63 *Meligethes* s.l. [*Meligethes* s.str. +  *Odonthogethes*] species and on selected outgroup species, including *Brassicogethes*.  **Characters**  _______________________________________________________________________________________   \| **1. Body dorsal color** \| \| --- \| \| 1. Variably colored, but never uniformly colored with violet to bluish reflections, (1) uniformly colored   with more or less distinct violet to bluish reflections  **2. Body dorsal color** \| \| 1. Variable, but never uniformly metallic green to olivaceous, or markedly bicolored with completely   orange-yellowish pronotum contrasting with blackish to bluish elytra and scutellum; or, if so, then  pronotum at least with infuscate darker discal maculations, (1) pronotum unicolorous yellowish to  orange (never with discal infuscated darker maculations), markedly contrasting with blackish to bluish  elytra and scutellum, (2) uniformly metallic green to olivaceous  **3. Body dorsal color (if tarsal claws toothed)** \| \| (0) Variably colored, but never with pronotum markedly paler and yellowish at sides, contrasting with an  uniformly much darker discal area, (1) pronotum markedly paler and yellowish at sides, contrasting with  an uniformly much darker discal area  **4. Body dorsal color (if tarsal claws not toothed)** \| \| (0) Variable, but never with reddish to pale brown pronotum always exhibiting more or less distinct darker  infuscated discal maculations, (1) pronotum reddish to pale brown, always exhibiting more or less distinct  darker infuscated discal maculations  **5. Body dorsal color (if tarsal claws toothed)** \| \| (0) Variably colored, but never uniformly yellowish to orange, without infuscate darker discal pronotal  area, (1) uniformly yellowish to orange, without infuscate darker discal pronotal area, (2) brown to  dark brown with more or less symmetrical yellowish maculations on sides of pronotum and elytra  **6. Body dorsal color and pubescence (if tarsal claws not toothed)** \| \| (0) Head, pronotum and elytra variably colored, but never completely blackish and not combined with  very long silver or golden pubescence on posterior half of pronotum, contrasting with much shorter  elytral pubescence, (1) dorsal color completely blackish and combined with very long silver or golden  pubescence on posterior half of pronotum, contrasting with the markedly shorter elytral pubescence  **7. Dorsal pubescence color (if tarsal claws toothed)** \| \| (0) Uniformly colored, (1) bicolored, at least partially darker on elytra, or with darker pronotal spots,  or with creamy longitudinal elytral stripes  **8. Dorsal pubescence color (if bicolorous)** \| \| (0) At least partially darker on elytra, (1) with at least traces of creamy longitudinal elytral stripes  **9. Dorsal pubescence length of elytra (if tarsal claws not toothed)** \| \| (0) Each individual elytral hair shorter, at most ca. 0.5-0.6× as long as tarsal claw, or less, (1) each  individual elytral hair markedly longer, ca. 1.0-1.1× as long as tarsal claw  **10. Dorsal pubescence length** \| \| (0) Uniform throughout the whole pronotum, scutellar shield and elytra, (1) much longer on posterior  portion of pronotum and scutellar shield, shorter on elytra  **11. Anterior edge of clypeus (if tarsal claws toothed)** \| \| (0) Truncate, (1) slightly to markedly emarginated  **12. Temples behind eyes (posterolateral ventral view)** \|   (0) Without any pit, (1) with distinct elliptic pits  **13. Temples behind eyes (if pits present)** |
| --- | --- | --- | --- | --- | --- | --- | --- | --- | --- | --- | --- | --- |
| (0) Pit placed inside distal posterior portion of the antennal grooves, (1) pit placed outside the  antennal grooves  **14. Circum-ocular furrows (occipital sulci on dorsal view of the head)** |
| (0) Present, (1) absent  **15. Circum-ocular edge on head (surrounding the ventral posterior portion of eyes)** |
| (0) Distinct, roughly "C"-shaped, (1) absent  **16. Inner edges of the antennal ventral furrows (surrounding the inner ventral portion of eyes)**   1. Markedly convergent posteriorly, parallel-sided anteriorly, (1) almost parallel-sided throughout their   length, or even divergent posteriorly  **17. Inner edges of the antennal ventral furrows (if not convergent posteriorly)**   1. almost parallel-sided throughout their length, (1), slightly divergent posteriorly   **18. [Male] 5^th^ antennomere (ratio L05J/W05J)** |
| (0) Ratio L05J/W05J <2.2, (1) ratio L05J/W05J => 2.2  **19. 3^rd^ and 2^nd^ antennomeres (ratio L03J/L02J)** |
| (0) Ratio L03J/L02J > 0.9, <= 1.4, (1) ratio L03J/L02J > 1.4, (2) ratio L03J/L02J <= 0.9  **20. Number of hairs along the distal edge of each side of the 9^th^ antennomere (if front tibiae not**  **exhibiting outwards long isolated teeth)** |
| (0) Ca. 12-16, (1) ca. 6-9  **21. Antennal club (ratio length/width)**  0) slender, > 1.30× as long as wide, (1) shorter and wider, slightly more compact, < 1.25×, >1.15× as long  as wide, (2) markedly shorter, wider, and more compact, < 1.10-1.15× as long as wide  **22. Shape of the combined outer edges of the antennal grooves on ventral side of the head**  (0) nearly parallel-sided throughout their entire length, or slightly and regularly divergent posteriorly  (1) More or less markedly convergent at least posteriorly |
| **23. Shape of the combined outer edges of the antennal grooves on ventral side of the head (if parallel-**  **sided or divergent posteriorly)**  (0) nearly parallel-sided throughout their entire length, (1) slightly and regularly divergent posteriorly  **24. Pronotal posterior angles** |
| (0) not projecting backwards, (1) only very slightly projecting backwards, (2) Distinctly projecting backwards  **25. Pronotal shape** |
| (0) Not trapezoidal, lateral sides more or less curved, (1) distinctly trapezoidal, lateral sides almost  straight at least in posterior two-thirds  **26. Outer sides of the dorsal portion of pronotum** |
| (0) Very narrowly bordered, (1) more or less widely flattened  **27. Shape of the microsetae along the posterior edge of pronotum** |
| (0) Styloids (bifid or trifid toward their apex), (1) hydroids (multifid from the middle towards their  apex)  **28. Length of the pubescence on the scutellar shield and elytra (if tarsal claws not toothed)** |
| (0) Nearly the same, (1) distinctly longer on scutellar shield, shorter on elytra  **29. Pronotal-prosternal sutures (notosternal sutures of the prothorax)** |
| (0) Not raised, (1) distinctly raised anteriorly, although oblitering posteriorly  **30. Shape of prosternal process** |
| (0) Clearly delimiting its "ventral" nearly flat portion, (1) without a delimited "ventral" portion  **31. Shape of prosternal process (apex)** |
| (0) More or less acutely pointed distad, (1) obtusely rounded or subtruncate distad  **32. Shape of prosternal process (apex; if not obtusely rounded or subtruncate)** |
| (0) Shortly and widely pointed distad, (1) acutely and narrowly pointed distad  **33. Punctation of anterior middle portion of prosternum** |
| (0) With distinct and more or less deep punctures, (1) without visible punctures, or with very  shallow ones  **34. Elytral discal surface between punctures**  (0) Smooth and shining, or nearly so, (1) more or less distinctly microreticulated and duller  **35. Elytral surface (if tarsal claws toothed)** |
| (0) Without distinct traces of transversal strigosity, (1) with more or less distinct traces of transversal  strigosity only towards elytral sides, (2) elytra markedly transversely strigose on most of their  surface  **36. Elytra at humeral bulge**  (0) Without any traces of a longitudinal stria prolonged posteriorly, (1) with barely distinct traces of a  longitudinal stria, at least in its middle portion, (2) with distinct traces of a longitudinal stria prolonged  posteriorly  **37. [Female] elytral tip** |
| (0) Separately rounded or subtruncate, (1) prolonged into a blunt distal lobe  **38. Posterior edge of metaventrite**  (0) Posterior angles not prolonged backward, (1) posterior angles slightly prolonged backward  **39. Posterior edge of metaventrite (if not prolonged backward)**  (0) Outer posterior angles widely obtuse and blunt, (1) outer posterior angles well distinct and only  slightly obtuse  **40. Distal portion of metepimera**  (0) Never approaching the “axillary line” of the first abdominal ventrite, (1) nearly approaching or even  crossing the “axillary line” of the first abdominal ventrite  **41. Distal portion of metepimera (if approaching or crossing the first abdominal ventrite’s**  **“axillary line”)**   1. Nearly approaching the “axillary line” of the first abdominal ventrite, (1) distinctly crossing the “axillary   line of the first abdominal ventrite  **42. Outer edge of the front tibia** |
| (0) With a series of small and regular teeth, (1) with a series of large and irregular teeth,  (2) without teeth  **43. Outer edge of the front tibia**   1. Tegumental projections between teeth triangular and partially covering the base of each interdenticular   spicula, (1) tegumental projections between teeth barely developed or even absent, not covering the  base of each interdenticular spicula  **44. Tarsal claws** |
| (0) Not toothed at base, (1) more or less distinctly and sharply toothed at base  **45. Tarsal claws (if toothed)** |
| (0) Obtusely and minutely toothed at base, (1) strongly and acutely toothed at base  **46. Metatibiae (ratio WPTI/LPTI)** |
| (0) Short, > 0.30, (1) Long, <= 0.30  **47. Axyllary triangular spaces close to proximal base at both sides of the first abdominal ventrite** |
| (0) Present, (1) almost absent  **48. Axyllary triangular spaces (if present) close to proximal base at both sides of the first abdominal**  **ventrite** |
| (0) Larger, slightly larger than metatrochanter, (1) smaller, smaller than metatrochanter  **49. Semi-circular arched impressions on both sides of the proximal basal portion of the last abdominal**  **ventrite** |
| (0) Present, (1) absent  **50. Semi-circular arched impressions (if present) on both sides of the proximal basal portion of the last**  **abdominal ventrite** |
| (0) Larger, their diameter nearly as long as eyes, (1) smaller, their diameter markedly shorter than  eyes  **51. Apex of pygidium**   1. Simple, rounded, in both sexes not prolonged distad in a more or less sharp and prominent lobe, (1)   at least in one of the two sexes prolonged distad in a more or less sharp and prominent lobe  **52. Apex of pygidium** **(if lobed at least in one of the two sexes)**   1. Always simple, rounded, in males, prolonged distad in a more or less sharp and prominent lobe in   females, (1) always simple, rounded, in females, prolonged distad in a more or less sharp and  prominent lobe in males, (2) in both sexes prolonged distad in a more or less sharp and  prominent lobe  **53. [Male] inner edge of distal excision of parameres in dorsal view (if tarsal claws not toothed)** |
| (0) Without distinct angulosity, (1) with distinct angulosity or projection  **54. [Male] development and relative length of the longest setae on distal portion of parameres in dorsal**  **view (ratio THLE/LETE)** |
| (0) THLE/LETE <= 0.11, (1) THLE/LETE => 0.14  **55. [Male] apex of the median lobe of aedeagus (if tarsal claws toothed)** |
| (0) Very minutely and narrowly incised distad, (1) not minutely and narrowly incised distad  **56. [Male] apex of the median lobe of aedeagus (if tarsal claws toothed)** |
| (0) Variably shaped, but not markedly narrowed at distal third and obtusely pointed distad,  (1) markedly narrowed at distal third and obtusely pointed distad  **57. [Male] apex of the median lobe of aedeagus (if tarsal claws not toothed)** |
| (0) Very minutely and narrowly incised distad, (1) not minutely and narrowly incised distad  **58. [Male] shape of the median lobe of aedeagus (if tarsal claws toothed)** |
| (0) Without chisel-shaped apex, (1) with chisel-shaped apex  **59. [Male] shape of the median lobe of aedeagus (if tarsal claws not toothed)** |
| (0) Without chisel-shaped apex, (1) with chisel-shaped apex  **60. [Male] shape of the median lobe of aedeagus (if tarsal claws toothed)** |
| (0) Ratio LEAE/WIAE = < 2, (1) ratio LEAE/WIAE > 2  **61. [Male] shape of the median lobe of aedeagus (if tarsal claws not toothed)** |
| (0) Ratio LEAE/WIAE = < 2, (1) ratio LEAE/WIAE > 2  **62. [Male] sensillar conical setae along outer edge of aedeagus in dorsal view** |
| (0) Absent, (1) present  **63. [Male] main sclerites of aedeagal internal sac (flagellum) in lateral view** |
| (0) Arcuately rod-shaped or claw-shaped and smaller, (1) roughly "elephant's silhouette"-shaped  and larger  **64. [Female] combined apices of gonostyloids (ovipositor)** |
| (0) Convergently acutely pointed or obtusely rounded, (1) not acutely pointed or obtusely rounded  **65. [Female] combined apices of gonostyloids (ovipositor)** |
| (0) Not acutely ogive-shaped (pointed arch), (1) acutely ogive-shaped (pointed arch)  **66. [Female] combined apices of gonostyloids (ovipositor; if tarsal claws not toothed)** |
| (0) More or less regularly pointed, or rounded distad, (1) incised, arcuately truncated, or  truncated distad  **67. [Female] combined apices of gonostyloids (ovipositor; if tarsal claws toothed)** |
| (0) More or less regularly pointed, or rounded distad, (1) incised, arcuately truncated, or  truncated distad  **68. [Female] outer portion of each individual apex of gonostyloids (ovipositor) after the insertion of the**  **styli** |
| (0) Never indented, (1) minutely to markedly indented  **69. [Female] each individual apex of gonostyloids (ovipositor) just before the insertion of the styli** |
| (0) Without a marked outer angulosity, (1) with a marked outer angulosity  **70. Larval host plants** |
| (0) Dicots, (1) Monocots  **71. Larval host plants (if Dicots)** |
| (0) Other families than Rosaceae or Brassicaceae, (1) Rosaceae, (2) Brassicaceae  **72. Larval host plants (if Rosaceae)** |
| (0) Rosoideae, (1) Spiraeoideae  **73. Larval host plants (if Rosoideae)** |
| (0) *Rosa* spp., (1) *Rubus* spp.  **74. Larval host plants (if Spiraeoideae)** |
| (0) *Prunus* spp., (1) *Malus* spp., *Crataegus* spp., *Pyracantha* spp., *Photynia* spp., (2) *Sorbaria* spp. |
