# Supplementary material for: Rosaceae, Brassicaceae and pollen beetles: exploring relationships and evolution in an anthophilous beetle lineage (Nitidulidae, Meligethes-complex of genera) using an integrative approach
Source: Front Zool. 2021 Mar 6;18:9. doi: 10.1186/s12983-021-00390-4 (PMC7936458; doi:10.1186/s12983-021-00390-4)
Supplement: Supplementary file 4 — Additional file 4 The Bayesian tree of Meligethes-complex on analyses of the mitochondrial COI gene. The posterior probabilities exceeding 50% are shown at nodes. [file 12983_2021_390_MOESM4_ESM.pdf]

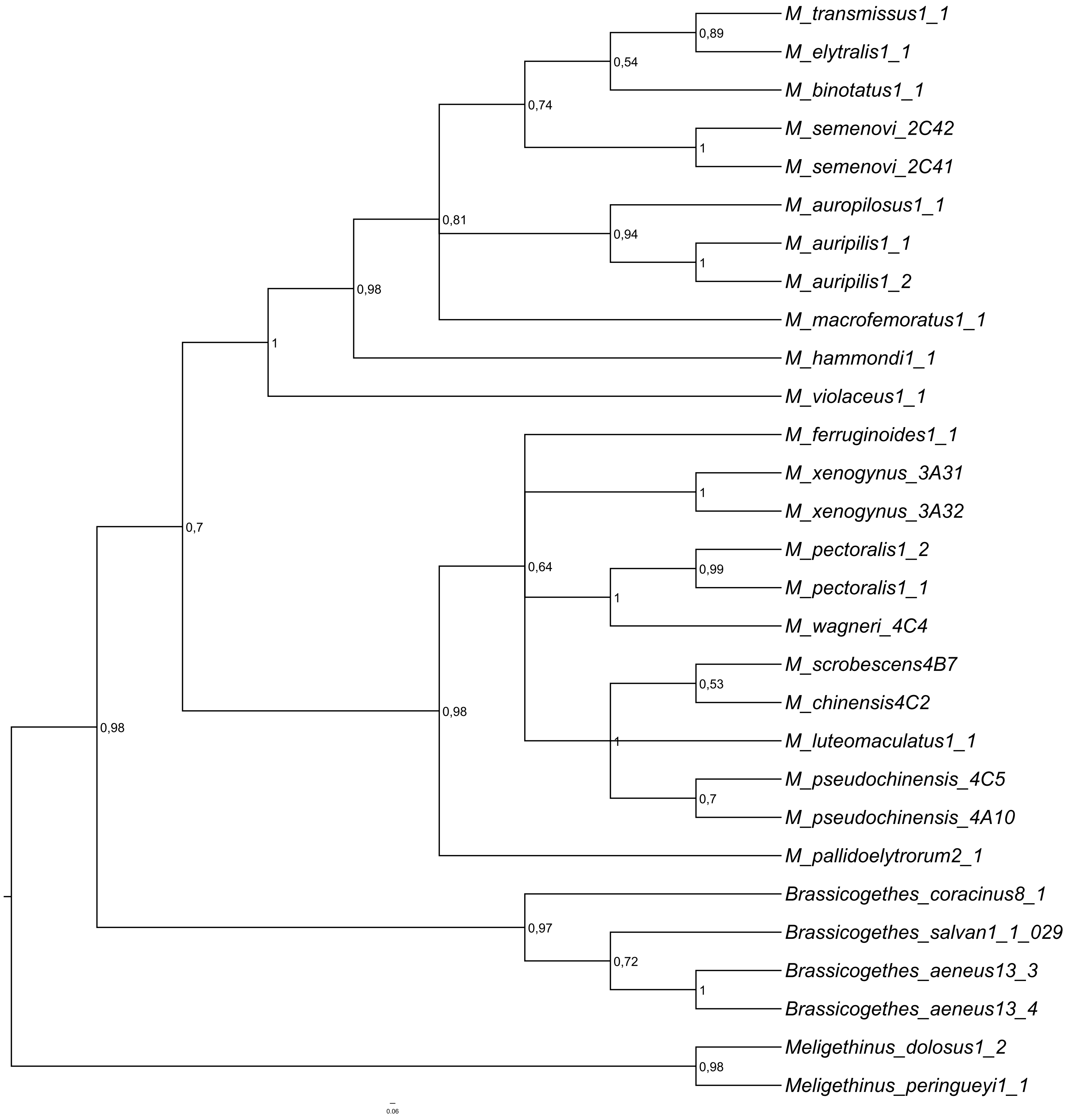

The Bayesian tree of *Meligethes*-complex on analyses of the mitochondrial COI gene.
The posterior probabilities exceeding 50% are shown at nodes.
