# Supplementary figures and images for: Rosaceae, Brassicaceae and pollen beetles: exploring relationships and evolution in an anthophilous beetle lineage (Nitidulidae, Meligethes-complex of genera) using an integrative approach
Source: Front Zool. 2021 Mar 6;18:9. doi: 10.1186/s12983-021-00390-4 (PMC7936458; doi:10.1186/s12983-021-00390-4)

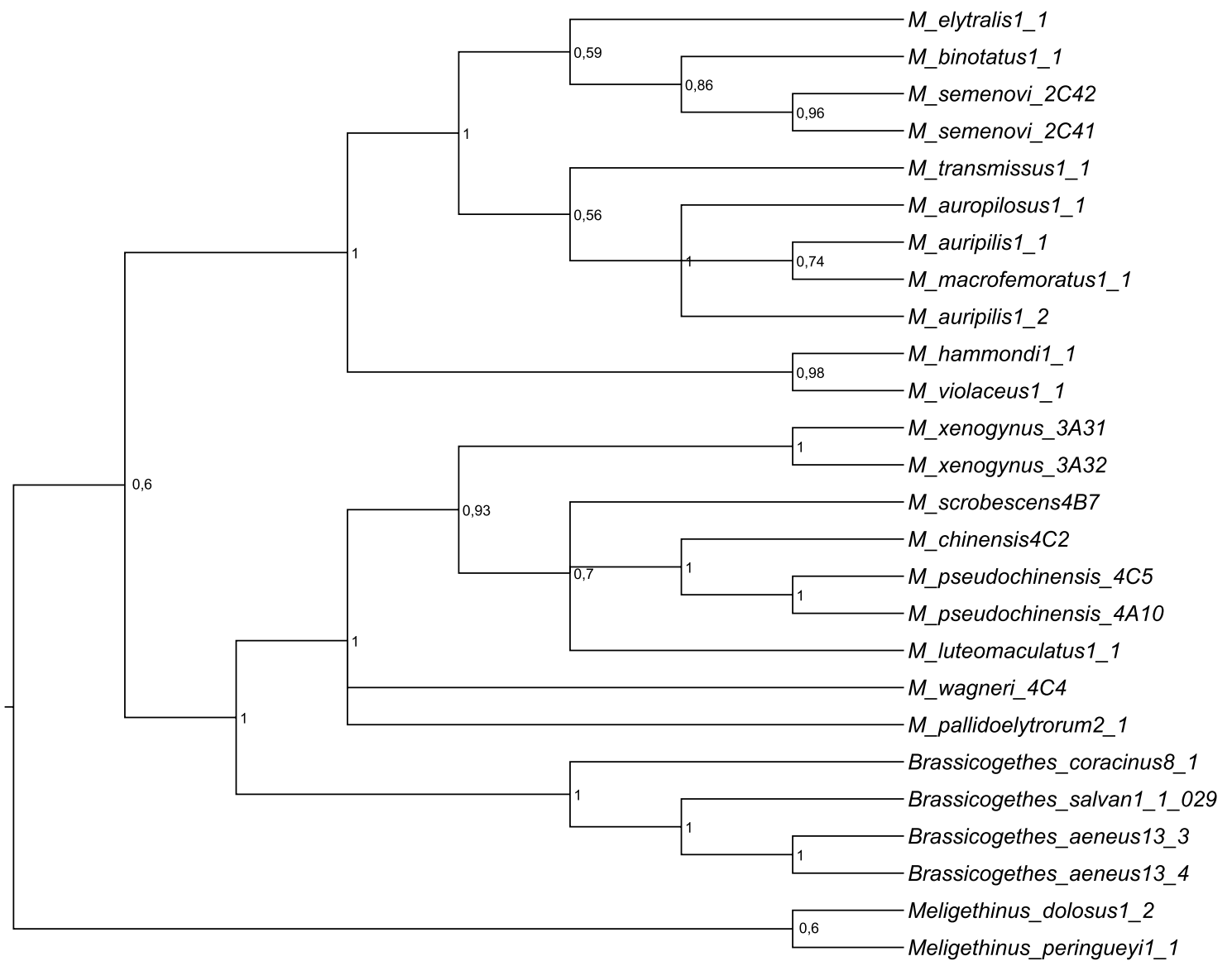

The Bayesian tree of *Meligethes*-complex on analyses of the nuclear CAD gene.

Supplement: Supplementary file 6 — Additional file 6 The Bayesian tree of Meligethes-complex on analyses of the nuclear CAD gene. The posterior probabilities exceeding 50% are shown at nodes. [file 12983_2021_390_MOESM6_ESM.pdf]

- *Meligethes s.l.*
- *Meligethes s.str.*
- *Odonthogethes*
- *Brassicogethes*
- *Meligethinus*

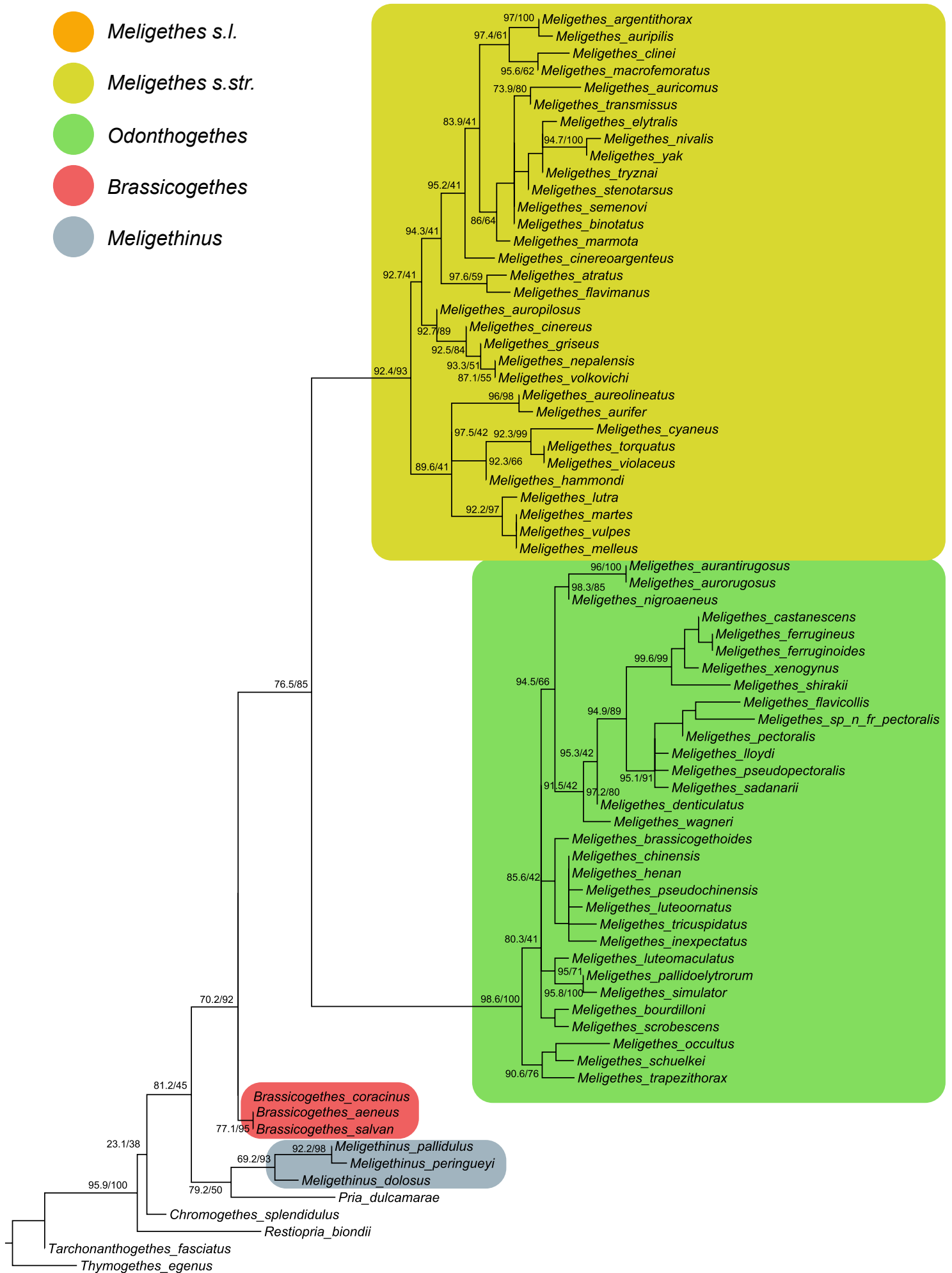

Supplement: Supplementary file 8 — Additional file 8 The Maximum Likelihood (ML) tree of Meligethes-complex based on morphological data, performed by IQ-TREE using default settings. [file 12983_2021_390_MOESM8_ESM.pdf]
