# Supplementary material for: Rosaceae, Brassicaceae and pollen beetles: exploring relationships and evolution in an anthophilous beetle lineage (Nitidulidae, Meligethes-complex of genera) using an integrative approach
Source: Front Zool. 2021 Mar 6;18:9. doi: 10.1186/s12983-021-00390-4 (PMC7936458; doi:10.1186/s12983-021-00390-4)
Supplement: Supplementary file 7 — Additional file 7 The Bayesian tree of Meligethes-complex based on combined molecular and morphological dataset. The posterior probabilities exceeding 50% are shown at nodes. [file 12983_2021_390_MOESM7_ESM.pdf]

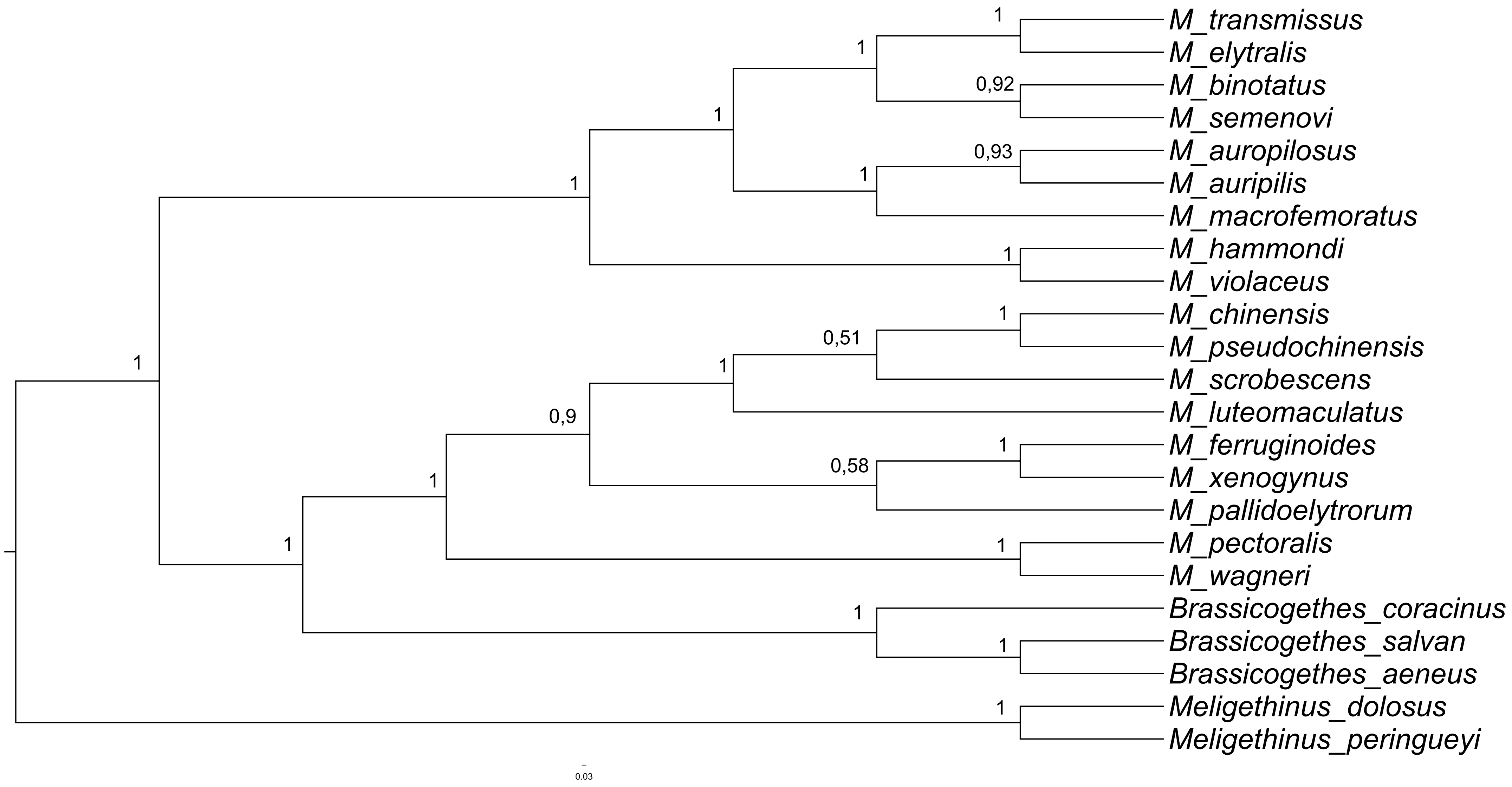

The Bayesian tree of Meligethes-complex based on combined molecular and morphological dataset.
The posterior probabilities exceeding 50% are shown at nodes.
